# SlSEC1- and SlSPY-mediated *O*-glycosylation stabilizes the transcription factor SlNOR to promote tomato fruit ripening

**DOI:** 10.1093/plcell/koag144

**Published:** 2026-05-14

**Authors:** Yu-Di Wu, Ruo-Han Ou, Can Yang, Tong-Hao Cui, Qian-Yu Wang, Yu-Yang Mei, Jia-Fei Qian, Yi-Long Liu, Yuan-Jiang Pan, Zhi-Ping Deng, Qing-Qiu Gong, Jing Li, Zhen-Yu Qi, Yan-Na Shi, Donald Grierson, Bo Zhang, Kun-Song Chen, Xian Li, Xiao-Yong Zhao

**Affiliations:** Zhejiang Key Laboratory of Horticultural Crop Quality Improvement, Zhejiang University, Hangzhou 310058, P.R. China; Zhejiang Key Laboratory of Horticultural Crop Quality Improvement, Zhejiang University, Hangzhou 310058, P.R. China; Zhejiang Key Laboratory of Horticultural Crop Quality Improvement, Zhejiang University, Hangzhou 310058, P.R. China; Zhejiang Key Laboratory of Horticultural Crop Quality Improvement, Zhejiang University, Hangzhou 310058, P.R. China; Zhejiang Key Laboratory of Horticultural Crop Quality Improvement, Zhejiang University, Hangzhou 310058, P.R. China; Zhejiang Key Laboratory of Horticultural Crop Quality Improvement, Zhejiang University, Hangzhou 310058, P.R. China; Zhejiang Key Laboratory of Horticultural Crop Quality Improvement, Zhejiang University, Hangzhou 310058, P.R. China; Zhejiang Key Laboratory of Horticultural Crop Quality Improvement, Zhejiang University, Hangzhou 310058, P.R. China; Department of Chemistry, Zhejiang University, Hangzhou 310058, P.R. China; Institute of Virology and Biotechnology, Zhejiang Academy of Agricultural Sciences, Hangzhou 310021, P.R. China; School of Life Sciences and Biotechnology, Shanghai Jiao Tong University, Shanghai 200240, P.R. China; College of Chemical and Biological Engineering, Zhejiang University, Hangzhou 310058, P.R. China; Agricultural Experiment Station of Zhejiang University, Zhejiang University, Hangzhou 310058, P.R. China; Zhejiang Key Laboratory of Horticultural Crop Quality Improvement, Zhejiang University, Hangzhou 310058, P.R. China; Zhejiang Key Laboratory of Horticultural Crop Quality Improvement, Zhejiang University, Hangzhou 310058, P.R. China; Plant and Crop Sciences Division, School of Biosciences, University of Nottingham, Loughborough LE12 5RD United Kingdom; Zhejiang Key Laboratory of Horticultural Crop Quality Improvement, Zhejiang University, Hangzhou 310058, P.R. China; Zhejiang Key Laboratory of Horticultural Crop Quality Improvement, Zhejiang University, Hangzhou 310058, P.R. China; Zhejiang Key Laboratory of Horticultural Crop Quality Improvement, Zhejiang University, Hangzhou 310058, P.R. China; Zhejiang Key Laboratory of Horticultural Crop Quality Improvement, Zhejiang University, Hangzhou 310058, P.R. China

## Abstract

*O*-glycosylation is a critical post-translational modification (PTM) that regulates protein function, yet its role in regulating plant transcription factors (TFs) remains poorly understood. Here, we report that *O*-glycosylation regulates NON-RIPENING (SlNOR), the master NAC TF controlling tomato (*Solanum lycopersicum*) fruit ripening. Using proteomic and interaction assays, we identified SlNOR as a substrate of 2 conserved nucleocytoplasmic *O*-glycosyltransferases: the *O*-GlcNAc transferase SlSEC1 and the *O*-fucosyltransferase SlSPY. We mapped 3 *O*-glycosylation sites (Thr93, Thr134, and Ser165) within the NAC domain of SlNOR. Biochemical assays suggested that *O*-glycosylation protects SlNOR from protein degradation. Accordingly, simultaneous mutagenesis of the 3 *O*-glycosylation sites reduced SlNOR protein stability and nuclear accumulation. Functionally, *O*-glycosylated SlNOR exhibited enhanced transcriptional activation of the ethylene biosynthesis genes (*SlACS2* and *SlACO1*), which was corroborated by its increased DNA-binding affinity in electrophoretic mobility shift assays. Genetic evidence from CRISPR/Cas9-generated mutants revealed that loss of SlSEC1 or SlSPY reduces ethylene production and delays ripening, while the *Slsec1-*1 *Slspy* double mutant displayed a cooperative ripening delay and severe growth defects. Collectively, our findings uncover a key PTM-based regulatory mechanism in which SlSEC1/SlSPY-mediated *O*-glycosylation enhances SlNOR stability and transcriptional activity, thereby coupling a master transcriptional regulator to ethylene biosynthesis for the control of fruit ripening.

## Teaser

SlSEC1 and SlSPY *O*-glycosylate the master ripening regulator SINOR, enhancing its transcriptional activity and stability to promote tomato fruit ripening.

## Introduction

Fruit ripening is a complex developmental process that determines fruit quality through dynamic changes in color, texture, and flavor. This process is orchestrated by a hierarchical regulatory network involving hormonal signals, transcription factors (TFs), and epigenetic modifications, etc. (Wang et al. 2023). In tomato (*Solanum lycopersicum*), a model for climacteric fruit, ripening is critically dependent on the hormone ethylene (Alexander and Grierson 2002; Klee and Giovannoni 2011; Giovannoni et al. 2017). Seminal studies of natural mutants such as *rin* (*ripening-inhibitor*) (Vrebalov et al. 2002), *nor* (*non-ripening*) (Martel et al. 2011), and *Cnr* (*Colorless non-ripening*) (Manning et al. 2006) identified master TFs that act upstream of ethylene synthesis to control the entire ripening program (Barry and Giovannoni 2007). Subsequent research has further clarified the intricate interplay between these TFs and ethylene in the ripening control network (Li et al. 2022).

Among these key regulators, SlNOR is a central NAC (NAM-ATAF1/2-CUC2) TF. The *nor* mutant fails to ripen and exhibits sharply reduced expression of key ripening-related genes, including those for ethylene biosynthesis (1-aminocyclopropanecarboxylate (ACC) synthase gene, *SlACS2*), carotenoid production (*SlGgpps2*), cell wall modification (*SlPL1*), and volatile synthesis (*SlLOXC*) (Giovannoni 2004; Gao et al. 2018). Proteomic comparisons between *nor* mutant and wild-type (WT) fruit revealed widespread downregulation of ethylene biosynthesis-related proteins, confirming its upstream role (Yuan et al. 2016). SlNOR directly binds the promoters of *SlACS2*, *SlACS4*, and ACC oxidase gene (*SlACO1*) to activate their transcription, thereby promoting ethylene production (Gao et al. 2020; Liu et al. 2024). Recent work has also linked SlNOR to epigenetic regulation through its activation of the DNA demethylase *SlDML2* gene, highlighting its multifunctional nature (Gao et al. 2022). Despite its importance, the regulation of SlNOR itself, particularly through post-translational modifications (PTMs), remains largely underexplored. While one study showed that oxidation of a methionine residue inhibits SlNOR DNA-binding activity (Jiang et al. 2020), the influence of glycosylation is unknown.

Protein glycosylation is a widespread PTM that fine-tunes protein function (Schjoldager et al. 2020). In the nucleus and/or cytoplasm, *O*-linked β-*N*-acetylglucosamine (*O*-GlcNAc) and fucose (*O*-fucose) modifications on serine (Ser)/threonine (Thr) residues are catalyzed by *O*-glycosyltransferases (Yang and Qian 2017; Aizezi et al. 2025). In animals, *O*-GlcNAc transferase (OGT) is the sole enzyme catalyzing *O*-GlcNAcylation and plays central roles in development, metabolism, and aging (Lee et al. 2021). In plants, its homologs SECRET AGENT (SEC) and SPINDLY (SPY) are thought to exert similar regulatory control. Studies in Arabidopsis have demonstrated their broad physiological importance. Notably, the *sec spy* double mutant is embryo lethal, indicating that OGT activity is essential for gametogenesis and embryogenesis (Hartweck et al. 2002). In addition, SEC and SPY regulate diverse developmental and signaling pathways, including roles in embryo development (Hartweck et al. 2006), gibberellin signaling (Robertson 2004; Silverstone et al. 2007), cytokinin signaling (Steiner et al. 2012), flowering time control (Tseng et al. 2004; Xing et al. 2018; Xu et al. 2019), and stress resistance (Qin et al. 2011; Xu et al. 2023a). Given the conserved link between OGT and aging in animals, and considering that fruit ripening represents a developmental transition toward senescence, it is plausible that SEC- and SPY-mediated *O*-glycosylation regulate ripening-associated pathways in plants. Indeed, SPY was recently shown to *O-*fucosylate the ethylene signaling component ETHYLENE INSENSITIVE 2 (EIN2) to promote tomato ripening (Xu et al. 2023b). However, the roles of SEC and SPY in fleshy fruit ripening remain limited, and their molecular mechanisms are not fully understood.

*O*-glycosylation plays a crucial role in cell signaling, transcription, and protein functional maintenance by modulating protein stability, subcellular localization, DNA binding, or intermolecular interactions (Jackson and Tjian 1988; Hart et al. 2011). About 15% (3,000 out of 20,000) of the proteins in the human proteome are *O*-GlcNAcylated (Ma et al. 2022). *O*-glycoproteomic studies in Arabidopsis and rice have identified numerous substrates of SEC and SPY, many of which are predicted to be involved in transcriptional regulation (Xu et al. 2017; Bi et al. 2023; Li et al. 2023). *O*-glycosylation of TFs such as TEOSINTE BRANCHED1/CYCLOIDEA/PROLIFERATING CELL FACTOR1 (TCP) (Steiner et al. 2012), CCCH zinc-finger protein SlC3H39 (Xu et al. 2023a), SPATULA (SPT) (Jiang et al. 2024), and AUXIN RESPONSE FACTOR (ARF) (Wang et al. 2025) is critical for their function in diverse plant processes. However, little is known about the *O*-glycosylation of TFs involved in fruit ripening. Given that SlNOR activity is essential for ripening, we are curious whether SlNOR is also post-translationally regulated by *O*-glycosylation.

Here, we investigate the role of *O*-glycosylation in regulating SlNOR function. We found that SlNOR is a substrate for both SlSEC1 and SlSPY. *O*-glycosylation at Thr93, Thr134, and Ser165 within SlNOR NAC domain is critical for its stability and transcriptional activity on ethylene biosynthesis gene promoters. Through genetic analysis, we establish that SlSEC1 and SlSPY function cooperatively as positive regulators of tomato fruit ripening. Our work thus identifies a regulatory layer where *O*-glycosylation of SlNOR controls the onset of fruit ripening.

## Results

### SlSEC1 and SlSPY cooperatively promote the onset of tomato fruit ripening

Given the parallels between fruit ripening and senescence, and OGT's role in animal aging, we hypothesized that SEC/SPY-mediated *O*-glycosylation regulates ripening—a premise we tested by applying specific inhibitors to immature green (IMG) tomato fruits. Pharmacological inhibition of SlSEC using OSMI-4 and SlSPY using TMC647055 choline salt delayed fruit ripening initiation by ∼3 d (Figure S1), suggesting both *O*-GlcNAc and *O*-fucose modifications contribute to the timing of ripening onset.

Two putative *SlSEC* genes (*SlSEC1* and *SlSEC2*) and one *SlSPY* gene were identified in the tomato genome using phylogenetic analysis (Figure S2a and Table S1). SlSEC1 and SlSEC2 share 89.15% amino acid identity and form a syntenic gene pair located within conserved collinear blocks between chromosome 05 and chromosome 09 (Figure S2b and File S1). Public RNA-seq data reported by Pattison et al. (2015) were retrieved using the TomExpress platform (https://tomexpress.gbfwebtools.fr/query) and *SlSEC2* and *SlSPY* transcripts accumulate at relatively high levels in seed tissues during early seed development, particularly in embryos at 4 days postanthesis (DPA), whereas *SlSEC1* expression is comparatively lower (Figure S2c). Interestingly, transcript analysis revealed that *SlSEC1* and *SlSPY* displayed broadly similar expression dynamics during fruit development and ripening and an obvious increase from breaker (Br) to 3 days after breaker (Br + 3) was observed, while *SlSEC2* displayed a different expression profile (Figure S2d). To assess functional contributions of SlSEC1 and SlSEC2 to ripening, we performed virus-induced gene silencing (VIGS) and found that silencing *SlSEC1* caused a more pronounced ripening inhibition than silencing *SlSEC2* (Figure S3). Thus, we selected *SlSEC1* and *SlSPY* for further genetic functional characterization.

**Figure 1:**
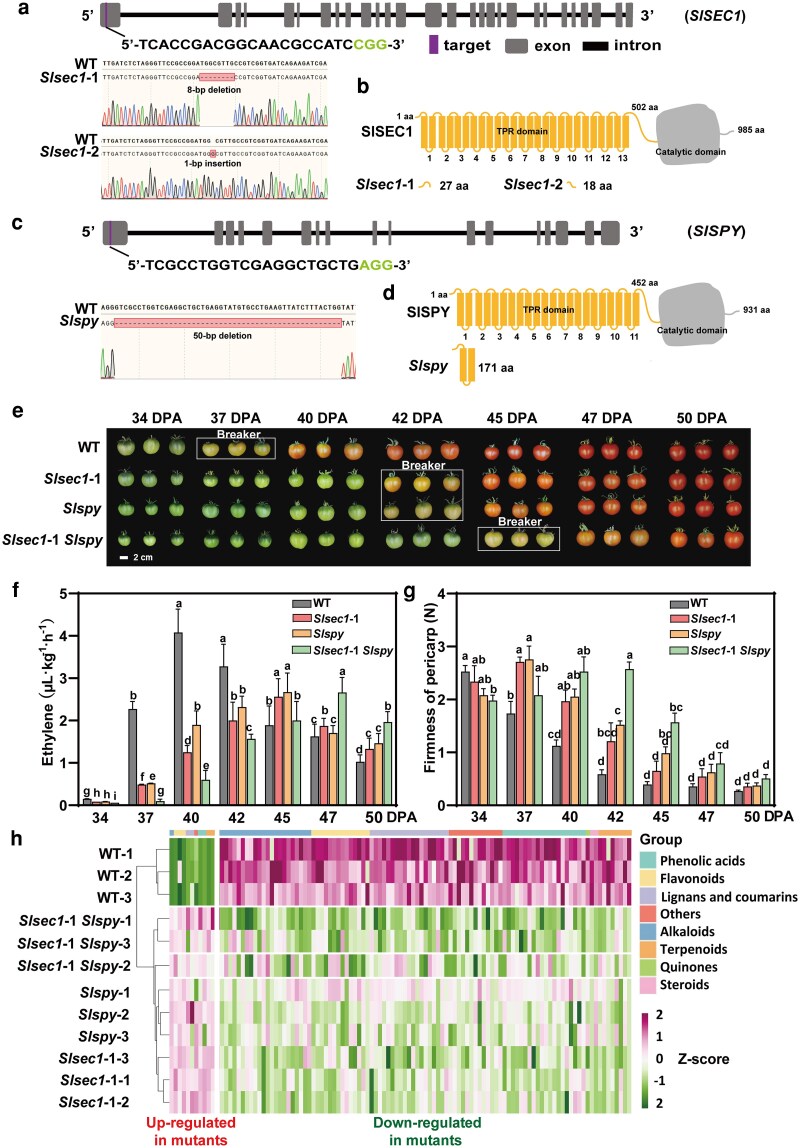
Genetic knockout of *SlSEC1* and *SlSPY* cooperatively delays tomato fruit ripening. a) Genomic structure of *SlSEC1* and CRISPR/Cas9-induced mutations in *Slsec1*-1 and *Slsec1*-2 alleles. Protospacer adjacent motifs (PAMs) are highlighted. b) Schematic of the SlSEC1 protein showing TPR and catalytic domains, and predicted truncations (27 aa in *Slsec1*-1 and 18 aa in *Slsec1*-2) in mutants. c) Genomic structure of *SlSPY* and CRISPR/Cas9-induced mutation in *Slspy* allele. PAMs are highlighted in green. d) Schematic of the SlSPY protein showing TPR and catalytic domains, and predicted truncations (171 aa) in *Slspy* mutant. e) Fruit ripening phenotypes of WT, *Slsec1-*1, *Slspy*, and *Slsec1-*1 *Slspy* double mutant tomatoes at indicated DPA. The breaker (Br) stage is highlighted with a white box. Images were acquired on separate dates as plants grew and were then digitally extracted for comparison. Scale bar, 2 cm. f, g) Ethylene production rate (f) and fruit firmness (g) of WT and mutant fruits at the indicated DPA stages. Values are mean ± SD (*n* = 3 independent biological replicates, each replicate containing 4 fruits). Different letters indicate statistically significant differences (*P* < 0.05, one-way ANOVA with Tukey's test). h) Heatmap showing the relative abundance of secondary metabolites in WT and mutant fruits at the 8 days after Br (Br + 8) stage. Metabolites are grouped by compound class. Hierarchical clustering was performed using Euclidean distance and complete linkage. The color scale represents Z-scores of metabolite abundance, indicating relative upregulation and downregulation in mutants, respectively.

To genetically confirm the role of *SlSEC1*, we generated CRISPR/Cas9 knockout mutants. Two independent *Slsec1* mutants were obtained: *Slsec1-*1 with an 8-bp deletion and *Slsec1-*2 with a 1-bp insertion, both resulting in frameshifts and producing severely truncated proteins (27 aa and 18 aa, respectively) (Fig. 1a and 1b). In parallel, we created a *Slspy* mutant harboring a 50-bp deletion, which is predicted to truncate the SlSPY protein, leaving only 1 tetratricopeptide repeat (TPR) domain and deleting the entire catalytic region (171 aa) (Fig. 1c and 1d).

To determine whether the generated mutants were enzymatically deficient, we examined *O*-glycosylation profiles in vivo. Equal protein loading was confirmed by Coomassie Brilliant Blue staining (Figure S4a). Western blotting with an anti-*O*-GlcNAc antibody (CTD110.6) revealed a reduction in SlSEC1-specific *O*-GlcNAcylated nuclear protein bands in *Slsec1-*1 and *Slsec1-*1 *Slspy* mutants compared with the WT (Figure S4b). Similarly, probing with the fucose-specific *Aleuria aurantia* lectin (AAL) showed a clear decrease or nearly loss of SlSPY-specific *O*-fucosylated nuclear protein bands in *Slspy* and *Slsec1-*1 *Slspy* mutants (Figure S4c), confirming the functional loss of the respective enzymes.

Fruit ripening was consistently delayed across all mutants. While WT fruit reached the Br stage at 37 DPA, *Slsec1-*1 and *Slspy* single mutants initiated ripening at 42 DPA, and the *Slsec1-*1 *Slspy* double mutant showed an even greater delay, turning breaker at 45 DPA, demonstrating a cooperative effect of SlSEC1 and SlSPY in controlling ripening time (Fig. 1e). This delay was confirmed in an independent *Slsec1-*2 allele (Figure S5), ruling out allele-specific effects. The phenotypic delay correlated with key ripening physiology: peak ethylene production occurred later (Fig. 1f) and fruit softening was significantly slower in all mutants (Fig. 1g).

Metabolic profiling further underscored the ripening defect. A widely targeted metabolomic analysis of fruits at Br + 8 stage showed a broad downregulation of metabolites in mutants versus WT, spanning phenolic acids, flavonoids, lignans, coumarins, alkaloids, terpenoids, quinones, and steroids (Fig. 1h;  Supplementary Data Set S1). The nearly identical downregulation patterns in *Slsec1-*1 and *Slspy* single mutants reinforced their cooperative action in ripening-associated metabolic reprogramming. Pearson correlation analysis of the metabolite profiles confirmed tight clustering of replicates within each genotype (correlation coefficients >0.89). Consistently, the metabolite profiles of *Slsec1-1* and *Slspy* mutants were highly correlated with each other, while the *Slsec1 Slspy* double mutant exhibited the lowest correlation with WT, further supporting the cooperative effect of SlSEC1 and SlSPY on ripening-associated metabolic reprogramming (Supplementary Data Set S1).

Beyond ripening, the mutants *Slsec1*-1, *Slspy*, and *Slsec1*-1 *Slspy* exhibited pleiotropic developmental defects, showing lower seed germination rates, reduced plant height, and decreased pollen number, with the most severe impairments observed in the *Slsec1-*1 *Slspy* double mutant (Figure S6). These findings indicate that SlSEC1 and SlSPY have broader roles in tomato development.

### SlSEC1 and SlSPY are required for ethylene sensitivity during postharvest ripening

**Figure 2:**
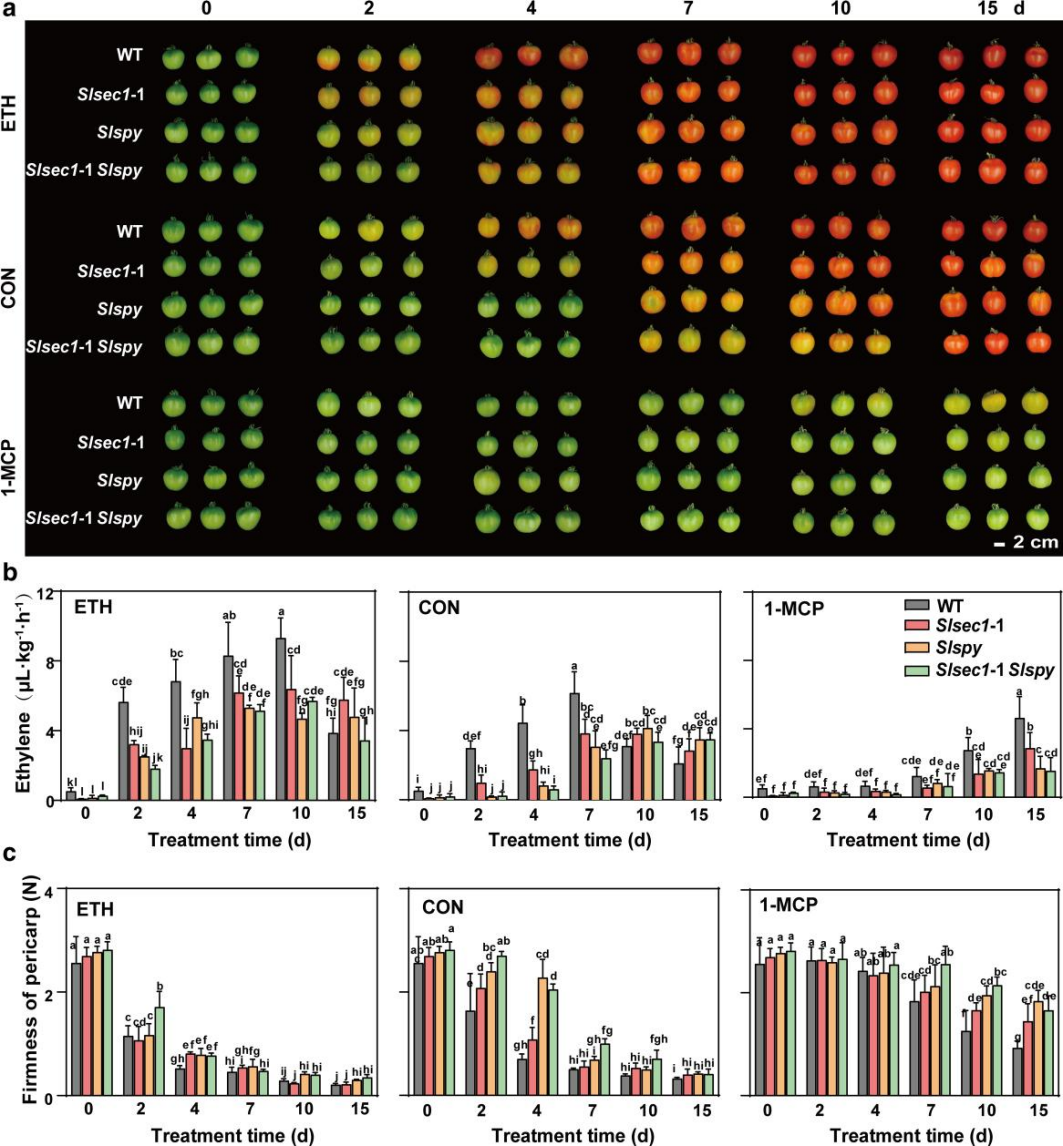
SlSEC1 and SlSPY are required for ethylene sensitivity during postharvest ripening. a) Postharvest ripening phenotypes of MG fruits from WT, *Slsec1-*1, *Slspy*, and *Slsec1-*1 *Slspy* mutant lines treated with air (CON), 100 ppm ethylene (ETH), or 10 ppm 1-MCP. Fruits were photographed at the indicated days after treatment. On each indicated day, all lines under different treatments were photographed using an identical background, and individual fruit images were then digitally extracted for comparison. Scale bar, 2 cm. b, c) Ethylene production rate (b) and fruit firmness (c) of treated fruits over the indicated time course. Values are mean ± SD (*n* = 3 independent biological replicates, each replicate containing 4 fruits). Different letters indicate statistically significant differences (*P* < 0.05, one-way ANOVA with Tukey's test).

To investigate the role of SlSEC1 and SlSPY in ethylene response, we treated mature green (MG) stage fruits with exogenous ethylene (ETH) or the ethylene action inhibitor 1-methylcyclopropene (1-MCP). As expected, 1-MCP delayed ripening in all genotypes. Under control (CON) conditions, WT fruits reached the breaker stage after 2 days (d) postharvest storage. In contrast, *Slsec1-*1 and *Slspy* single mutants required 4 to 6 d, and the *Slsec1-*1 *Slspy* double mutant required 7 d to reach the breaker stage (Fig. 2a). When treated with ETH, both *Slspy* and *Slsec1-*1 *Slspy* double mutants showed a perceptible insensitivity, while the *Slsec1-*1 mutant exhibited a more modest ripening delay compared with WT (Fig. 2a). Consistent with their ripening-delayed phenotype, both *Slspy* and *Slsec1-*1 *Slspy* mutants showed significantly reduced ETH production (Fig. 2b) and delayed softening (Fig. 2c) compared with the CON group. These results suggest that while both enzymes contribute to ETH sensitivity, SlSPY plays a more dominant role in modulating the fruit's responsiveness to ETH. Taken together, our findings show that SlSEC1 and SlSPY act upstream of ETH synthesis and modulate ETH sensitivity, thereby exerting dual control over tomato fruit ripening.

### The master ripening regulator SlNOR physically interacts with SlSEC1 and SlSPY

**Figure 3:**
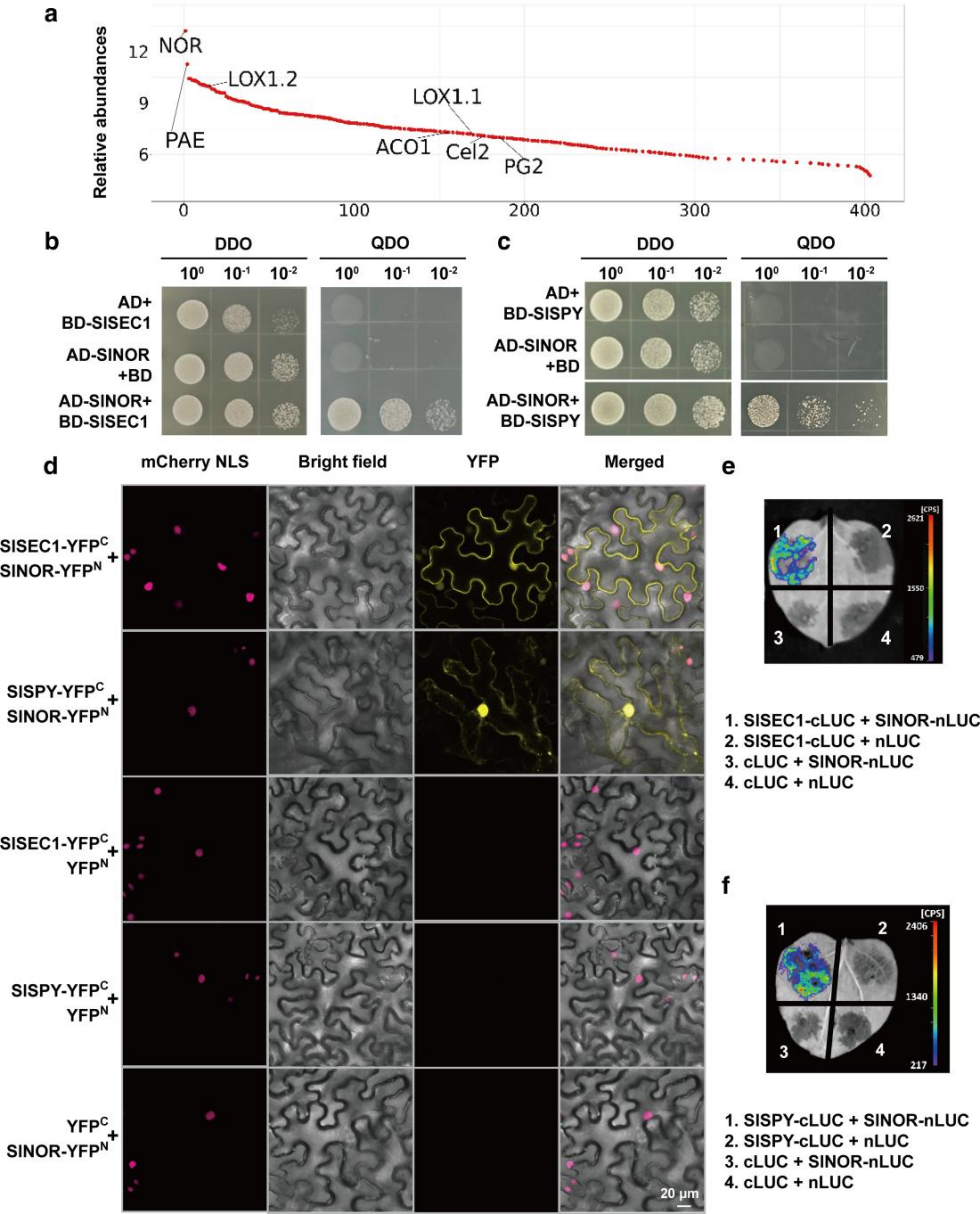
SlNOR physically interacts with SlSEC1 and SlSPY. a) Relative abundance of proteins copurified with the GST-tagged SlSPY TPR domain from fruit protein extracts. Ripening-related proteins such as SlNOR, pectin acetylesterase (SlPAE), lipoxygenases SlLOX1.1 and SlLOX1.2, 1-aminocyclopropanecarboxylate oxidase 1 (SlACO1), endoglucanase 2 (SlCel2), and polygalacturonase 2 (SlPG2) are marked. b, c) Y2H assays showing the interaction of SlNOR with SlSEC1 (b) and SlSPY (c). Yeast cultures were spotted in a 10-fold serial dilution series on nonselective (DDO) and selective (QDO) media. AD and BD empty vectors served as negative controls. d) BiFC assays in transgenic *N. benthamiana* leaves. Coexpression of SlNOR-YFP^N^ with SlSEC1-YFP^C^ or SlNOR-YFP^N^ with SlSPY-YFP^C^ reconstitutes the YFP signals, while coexpression of YFP^N^ with SlSEC1-YFP^C^ or SlSPY-YFP^C^ as well as coexpression of SlNOR-YFP^N^ with YFP^C^ show no detectable YFP signal. A nuclear mCherry fusion marks the nucleus. Scale bars, 20 μm. e, f) Firefly LCI assay showing the interaction between SlNOR-nLUC and SlSEC1-cLUC (e) or SlSPY-cLUC (f) in *N. benthamiana* leaves. Pseudocolor bar indicates signal intensity.

To identify the downstream targets of *O*-glycosyltransferases, we performed GST pull-down coupled with mass spectrometry (MS). Given the pronounced ripening delay in *Slspy* mutants (Fig. 1e) and prior evidence implicating SlSPY in ripening (Xu et al. 2023b), we used recombinant GST-tagged TPR domains of SlSPY (GST-nSlSPY) to capture interactors from tomato fruit protein extracts. Among 403 potential candidates, the master regulator SlNOR was the most abundant, suggesting a possible interaction with SlSPY (Fig. 3a;  Supplementary Data Set S2).

We confirmed this interaction using 3 independent methods: yeast two-hybrid (Y2H), bimolecular fluorescence complementation (BiFC), and luciferase complementation imaging (LCI). In the Y2H system, SlNOR interacted with both SlSEC1 (Fig. 3b) and SlSPY (Fig. 3c). BiFC and LCI assays performed in *Nicotiana benthamiana* leaves further confirmed these interactions in plant cells (Fig. 3d–f). In contrast, under identical experimental conditions, SlSEC2 did not interact with SlNOR in the BiFC assay. This specific interaction of SlNOR with SlSEC1 but not SlSEC2 provides another molecular rationale for our subsequent functional focus on SlSEC1. Subcellular localization analysis revealed that SlSEC1-GFP and SlSPY-GFP were present in both the nucleus and cytoplasm, with no detectable signal overlap with the plasma membrane marker syntaxin of plant 122 (SYP122) (Figure S7). The BiFC assay revealed distinct interaction patterns: the SlSPY-SlNOR pair exhibited a strong signal predominantly in the nucleus, while the SlSEC1-SlNOR pair showed a weaker signal localized to both the nucleus and cytoplasm (Fig. 3d). No signal was detected in the negative control combinations (Fig. 3d). Strong LCI signals were observed only when SlNOR-nLUC was coexpressed with SlSEC1-cLUC (Fig. 3e) or SlSPY-cLUC (Fig. 3f). Together, these assays firmly establish that SlNOR is a bona fide interactor of both SlSEC1 and SlSPY.

To assess the broader regulatory network, we also identified the SlSEC1 interactome. A total of 318 proteins overlapped between SlSEC1 and SlSPY interactors, with significant enrichment for cytoplasmic and nuclear proteins (Figure S8a, S8b). From the pool of overlapped interactors, we successfully identified several key ripening-related proteins, such as cell wall-modifying enzymes pectin acetylesterase (SlPAE), polygalacturonase 2 (SlPG2), and endoglucanase 2 (SlCel2), the ethylene-synthesizing enzyme 1-aminocyclopropanecarboxylate oxidase 1 (SlACO1), and the volatile-forming lipoxygenases SlLOX1.1 and SlLOX1.2, supporting the existence of a shared functional network (Fig. 3a;  Supplementary Data Set S2). Gene ontology (GO) analysis revealed that these common targets are involved in translation, protein folding, metabolism, and proteasome-mediated protein catabolism, underscoring the central role of *O*-glycosylation in cellular homeostasis (Figure S8c).

### SlNOR is *O*-glycosylated within its NAC DNA-binding domain

**Figure 4:**
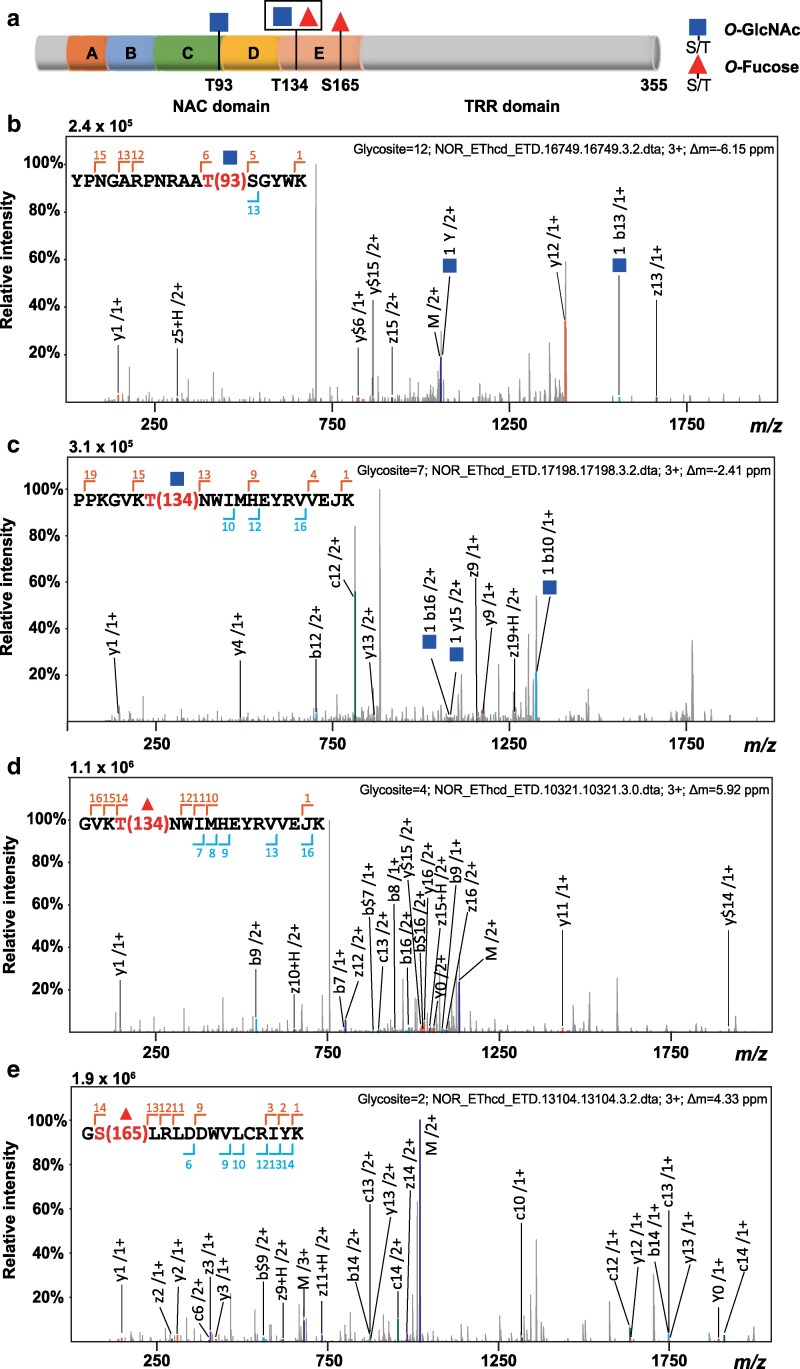
SlNOR is *O*-glycosylated within its NAC DNA-binding domain. a) Schematic of the SlNOR protein showing the N-terminal NAC domain (subdomains A–E) and C-terminal TRR. The identified *O*-glycosylation sites—Thr93 (within subdomain C) and Thr134 and Ser165 (both within subdomain E)—are indicated. b–e) Representative EThcD tandem mass spectra identifying *O*-glycosylation modifications at Thr93 (*O*-GlcNAc) (b), Thr134 (*O*-GlcNAc and *O*-fucose, dual occupancy) (c, d), and Ser165 (*O*-fucose) (e). The spectrum consists of the *O*-glycosite, EThcD mode, spectrum name, charge number of precursors, mass deviation, glycan composition, and peptide sequence with highlighted “S/T” indicating the *O*-glycosites. Peak annotation of the spectrum: Y denotes glycan ions with an intact peptide attached, b/y denotes naked peptide fragment ions, c/z denotes glycan-attached peptide fragment ions, and M denotes precursor ions. Squares and triangles denote *O*-GlcNAc and *O*-fucose modifications, respectively, in (a) to (e).

The physical interaction between SlNOR and the *O*-glycosyltransferases prompted us to investigate whether SlNOR is a substrate for *O*-glycosylation modification. We performed MS analysis on SlNOR-Flag transiently expressed in *N. benthamiana* leaves. This revealed that SlNOR undergoes *O*-glycosylation within its N-terminal NAC DNA-binding domain (16 to 180 aa). Using electron-transfer/higher-energy collision dissociation (EThcD), 3 specific *O*-glycosylation sites, Thr93, Thr134, and Ser165, were identified. Thr93 was modified with *O*-GlcNAc, Ser165 with *O*-fucose, and Thr134 showed dual occupancy with both *O*-GlcNAc and *O*-fucose (Fig. 4). The NAC DNA-binding domain of SlNOR is organized into 5 conserved subdomains (A–E) (Fig. 4a). Thr93 is located within subdomain C, while Thr134 and Ser165 reside within subdomain E (Fig. 4b–e;  Supplementary Data Set S3). To pinpoint the interaction domains, we conducted Y2H assays using truncated proteins. These assays showed that SlNOR binds to both SlSPY and SlSEC1 via its C-terminal transcriptional regulatory region (TRR, 181 to 355 aa), whereas the N-terminal region (1 to 180 aa) did not interact (Figure S9).

### *O*-glycosylation promotes SlNOR protein stability

**Figure 5:**
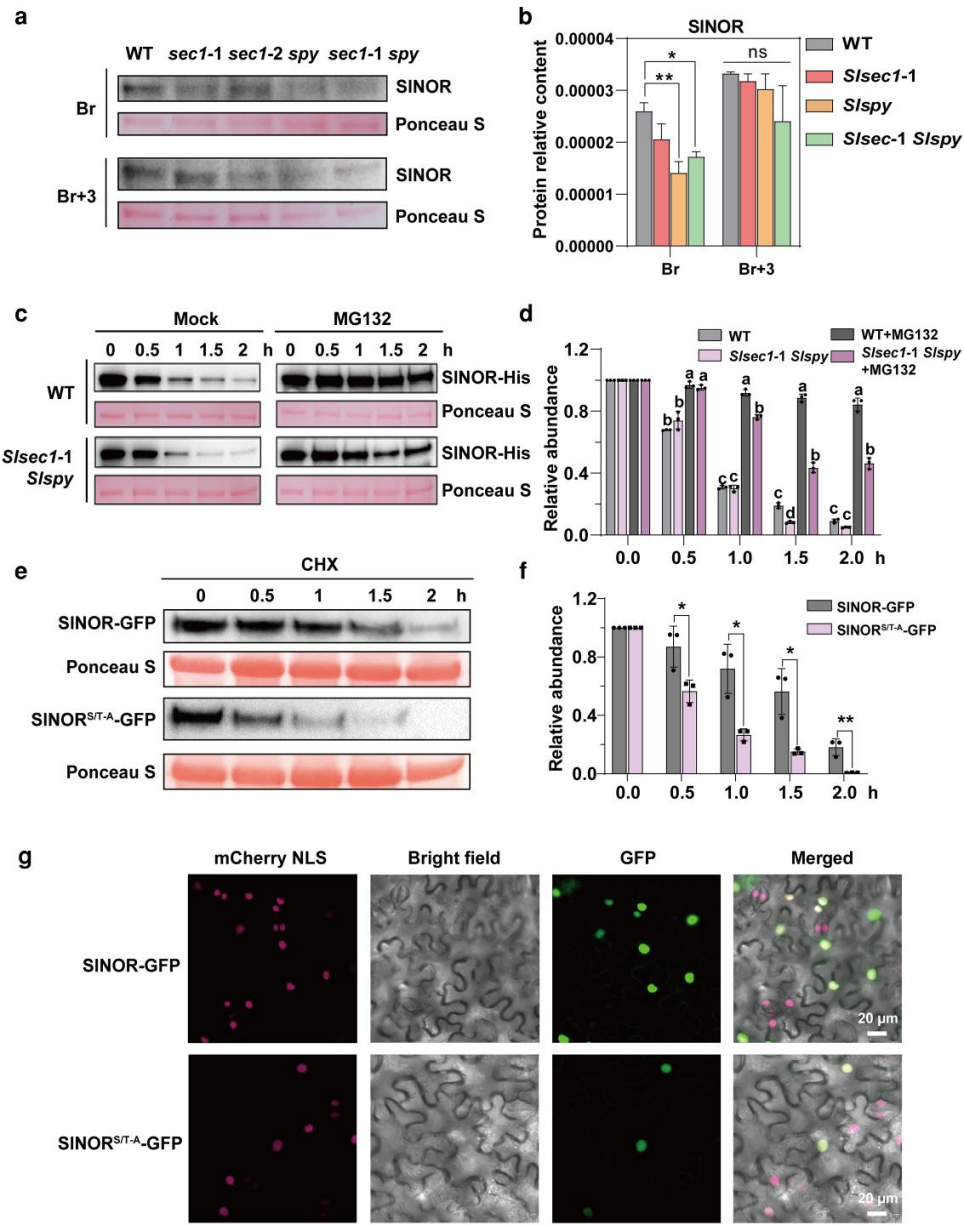
*O*-glycosylation promotes SlNOR protein stability. a) Immunoblot analysis of endogenous SlNOR protein levels in WT, *Slsec1-*1, *Slsec1-*2, *Slspy*, and *Slsec1*-1 *Slspy* mutant fruits at the Br and Br + 3 stages. Total proteins were immunoblotted with an anti-SlNOR antibody. Ponceau S staining is a loading control. b) Relative SlNOR protein abundance from proteomic analysis of WT and mutant fruits at the Br and Br + 3 stages. Values are mean ± SD. Different letters indicate statistically significant differences (*P < 0.05 and **P < 0.01, Student's *t*-test). c) Cell-free degradation assay of recombinant SlNOR-His incubated with WT or *Slsec1-*1 *Slspy* fruit protein extracts with/without the proteasome inhibitor MG132 (Z-Leu-Leu-Leu-al). Protein levels were detected by anti-His immunoblotting. d) Quantification of immunoblot signals from (c). Values are mean ± SD. Different letters indicate statistically significant differences (*P* < 0.05, one-way ANOVA with Tukey's test). e) CHX chase assay of SlNOR-GFP and SlNOR^S/T-A^-GFP in *N. benthamiana* leaves. Protein levels were detected by anti-GFP immunoblotting. Ponceau S staining shows equal loading in (a, c, e). f) Quantification of immunoblots from (e). Values are mean ± SD. Different letters indicate statistically significant differences (*P < 0.05 and **P < 0.01, Student's *t*-test). g) Subcellular localization and accumulation of SlNOR-GFP and SlNOR^S/T-A^-GFP in transgenic *N. benthamiana* leaves. A nuclear mCherry fusion marks the nucleus. Scale bar, 20 μm.

Next, we examined whether *O*-glycosylation affects SlNOR protein accumulation. *SlNOR* transcript levels remain largely unchanged, or even increased at Br stage in the mutants compared with WT (Figure S10), while SlNOR protein levels were substantially reduced in *Slsec1-*1, *Slsec1-*2, *Slspy*, and *Slsec1-*1 *Slspy* double mutant compared with the WT at the Br and Br + 3 stage (Fig. 5a). Consistently, quantitative proteomic analysis confirmed lower SlNOR protein abundance in mutant fruits (Fig. 5b), indicating that SlSEC1 and SlSPY are required for normal SlNOR accumulation during ripening.

To test if this reduction stems from altered protein stability, we performed cell-free degradation assays. Recombinant SlNOR-His protein showed greater degradation when incubated with protein extracts from *Slsec1-*1 *Slspy* mutant fruit compared with WT, and this accelerated degradation was largely suppressed by the proteasome inhibitor MG132 (Z-Leu-Leu-Leu-al) in WT protein extracts, while only partially inhibited in *Slsec1-*1 *Slspy* protein extracts (Fig. 5c and 5d), indicating both proteasome-dependent and proteasome-independent turnovers.

We confirmed these findings in vivo using a cycloheximide (CHX) chase assay in *N. benthamiana* leaves. While both SlNOR-GFP and SlNOR^S/T-A^-GFP levels declined after translation inhibition, SlNOR^S/T-A^-GFP was degraded much faster, becoming nearly undetectable after 1.5 h (Fig. 5e and 5f). Fluorescence microscopy revealed that while both proteins localized to the nucleus, the fluorescence intensity of SlNOR-GFP was much stronger than that of SlNOR^S/T-A^-GFP (Fig. 5g), indicating that *O*-glycosylation enhances SlNOR stability and nuclear accumulation without altering its subcellular destination. Collectively, these data suggest that *O*-glycosylation is required to maintain SlNOR protein stability during ripening.

### *O*-glycosylation of SlNOR is required for its transcriptional activity

**Figure 6:**
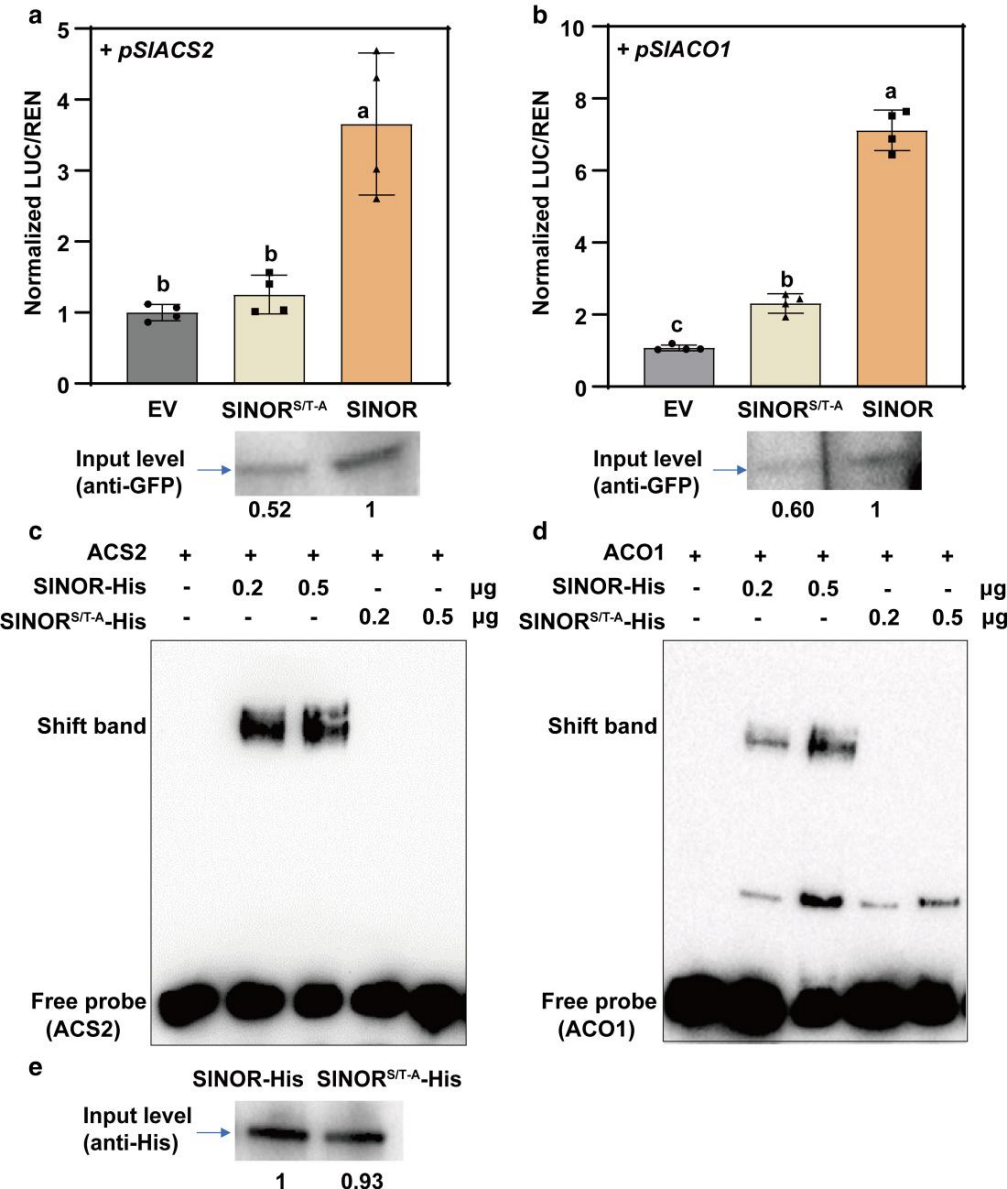
*O*-glycosylation of SlNOR is required for its transcriptional activity. a, b) Dual-luciferase reporter assays in *N. benthamiana* leaves showing transcriptional activation of the *SlACS2* (a) and *SlACO1* (b) promoters. WT SlNOR or the glycosylation-deficient mutant SlNOR^S/T-A^ were coexpressed with reporter constructs. Empty vector (EV) is a negative control. Values are mean ± SD (*n* = 4 independent biological replicates). Different letters indicate statistically significant differences (*P* < 0.05, one-way ANOVA with Tukey's test). Immunoblot analysis below each panel shows the protein abundance of SlNOR and SlNOR^S/T-A^. The presented reporter activities were normalized using input protein levels of SlNOR and SlNOR^S/T-A^ accordingly. c, d) EMSAs showing DNA-binding activity of recombinant SlNOR and SlNOR^S/T-A^ to probes derived from *SlACS2* (c) and *SlACO1* (d) promoters. Shifted complexes and free probes are indicated. e) Immunoblot confirming comparable input of His-tagged proteins used in EMSA.

We then investigated whether *O*-glycosylation affects SlNOR's function as a transcriptional activator of ethylene biosynthesis genes. ACC synthase gene *SlACS2* and ACC oxidase gene *SlACO1*, encoding key enzymes in ethylene synthesis, are well-established transcriptional targets of tomato SlNOR (Gao et al. 2020; Liu et al. 2024). In a dual-luciferase reporter assay in vivo, coexpression of WT SlNOR strongly activated both the *SlACS2* and *SlACO1* promoters, whereas the glycosylation-deficient mutant SlNOR^S/T-A^ showed a significantly reduced activation of both reporters (Fig. 6a and 6b). The presented reporter activities were normalized using input protein levels of SlNOR and SlNOR^S/T-A^ accordingly, confirming that the reduced activity of SlNOR^S/T-A^ was not due to expression differences (Fig. 6a and 6b). We further examined whether SlSEC1 and SlSPY influence SlNOR-mediated transcriptional activation. In dual-luciferase assays, expression of SlSEC1 or SlSPY alone did not activate the *SlACS2* or *SlACO1* promoters. Notably, coexpression of SlNOR with either SlSEC1 or SlSPY failed to synergistically enhance this activation, indicating that their physical interaction may not directly modulate SlNOR's transcriptional activity (Fig. 3; Figure S11). To determine whether *O*-glycosylation affects the DNA-binding capacity of SlNOR, we next performed electrophoretic mobility shift assays (EMSAs) using recombinant proteins in vitro. While recombinant SlNOR-His bound promoter fragments of *SlACS2* and *SlACO1*, SlNOR^S/T-A^-His failed to bind under identical conditions (Fig. 6c and 6d), indicating that 3 identified *O*-glycosylation sites (Thr93, Thr134, and Ser165) are critical for DNA-binding activity of SlNOR. Immunoblot confirming comparable inputs of His-tagged proteins used in EMSA (Fig. 6e). Nearly identical secondary structures of SlNOR-His and SlNOR^S/T-A^-His in circular dichroism (CD) suggested that the site mutations may not intrinsically induce improper folding (Figure S12).

### *O*-glycosylation of SlNOR enhances its DNA-binding capacity

**Figure 7:**
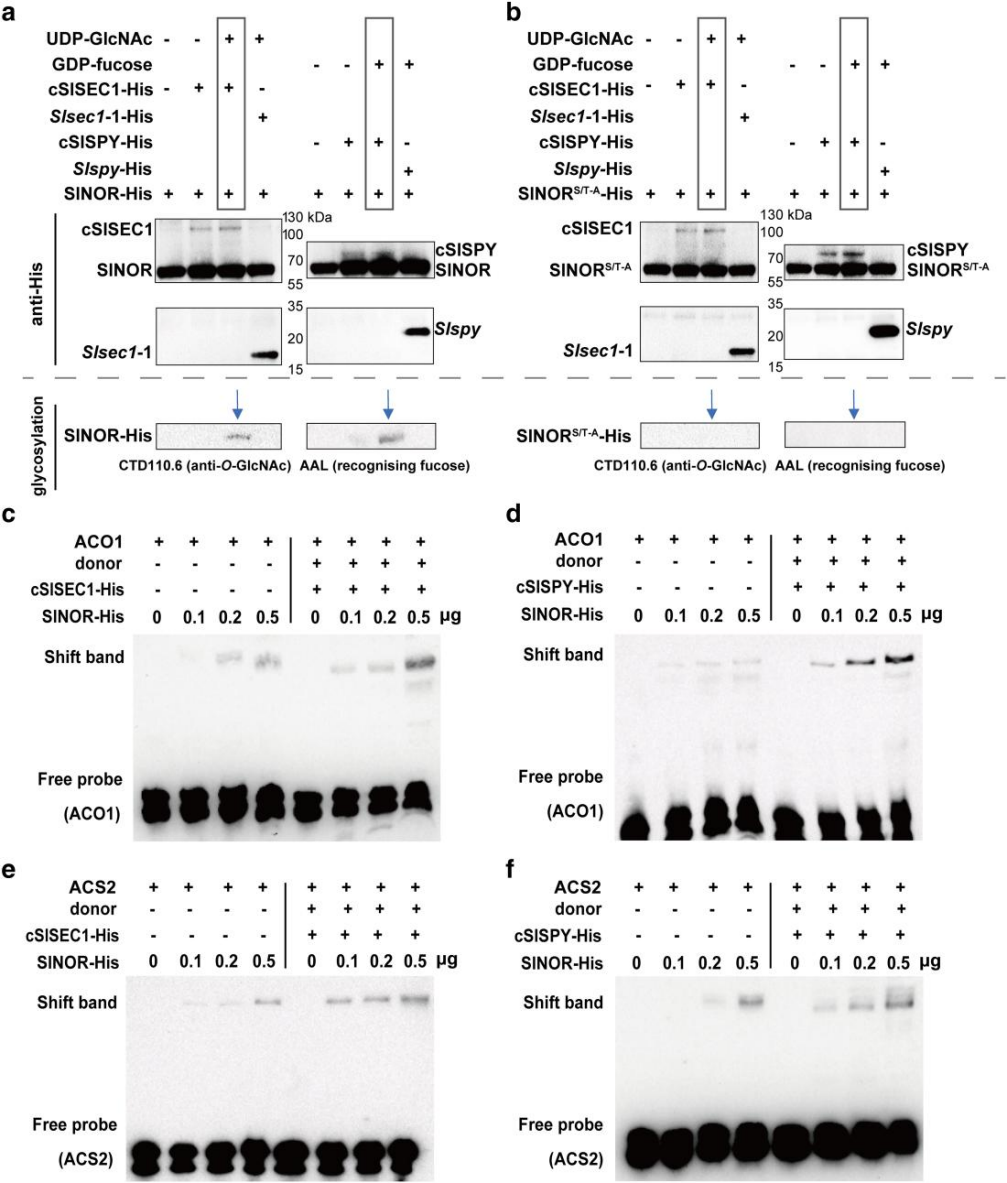
*O*-glycosylation of SlNOR enhances its DNA-binding capacity. a, b) In vitro *O*-glycosylation of recombinant SlNOR-His (a) and the glycosylation-deficient mutant SlNOR^S/T-A^-His (b). SlNOR-His or SlNOR^S/T-A^-His were incubated with UDP-GlcNAc or GDP-fucose in the presence of recombinant His-tagged catalytic domains of cSlSEC1 (360 to 964 aa and 5TPR plus catalytic domain) or cSlSPY (360 to 850 aa and 3TPR plus catalytic domain), respectively. Mutant version glycosyltransferase *Slsec1*-1-His or *Slspy*-His served as negative controls. Recombinant His-tagged proteins were confirmed using an anti-His antibody. *O*-glycosylation of SlNOR-His was detected by immunoblotting with anti-*O*-GlcNAc (CTD110.6) antibody or lectin blotting with AAL. c–f) EMSAs showing enhanced binding of recombinant SlNOR-His to biotin-labeled probes from the *SlACO1* (c, d) and *SlACS2* (e, f) promoters upon in vitro glycosylation. The addition of cSlSEC1 with UDP-GlcNAc (c, e) or cSlSPY with GDP-fucose (d, f) markedly increased DNA–protein complex formation. Shifted complexes and free probes are indicated.

To assess how *O*-glycosylation affects SlNOR transcriptional activity, we performed in vitro reconstitution of its *O*-GlcNAcylation and *O*-fucosylation. Immunoblotting with the anti-*O*-GlcNAc antibody CTD110.6 and lectin blotting with AAL confirmed the absence of both *O*-GlcNAcylation and *O*-fucosylation in bacterially expressed SlNOR-His and SlNOR^S/T-A^-His (lane 1, Fig. 7a and 7b). Incubation of SlNOR-His with the cSlSEC1-His (containing the 5TPR and catalytic domain) plus UDP-GlcNAc, or with the cSlSPY (containing the 3TPR and catalytic domain) plus GDP-fucose, efficiently restored *O*-GlcNAcylation or *O*-fucosylation, respectively (lane 3, Fig. 7a). In contrast, no glycosylation signal was detected for the glycosylation sites-mutated SlNOR^S/T-A^-His under any tested condition (Fig. 7b), confirming the specificity of the reactions.

Next, we examined whether glycosylation affects the DNA-binding capacity of SlNOR. EMSAs showed that in vitro glycosylation markedly enhanced the DNA-binding activity of SlNOR-His to the *SlACO1* and *SlACS2* promoters in a concentration-dependent effect (Fig. 7c–f; Figure S13a, S13c, and S13d). By contrast, the glycosylation sites-mutated SlNOR^S/T-A^-His failed to bind the probe regardless of glycosylation conditions (Figure S13b), indicating that the 3 identified *O*-glycosylation sites (Thr93, Thr134, and Ser165) within the NAC domain of SlNOR are essential for its DNA-binding capacity. Together, these results suggest that *O*-glycosylation potentiates SlNOR's transcriptional activity by strengthening its association with target gene promoters.

## Discussion

PTMs represent a crucial regulatory layer for fine-tuning protein function, enabling plants to dynamically integrate endogenous signals and coordinate complex developmental processes, including flowering, senescence, and organ maturation (Hart et al. 2011; Schjoldager et al. 2020). Fruit ripening is a particularly intricate developmental switch, requiring precise coordination of transcriptional programs and hormonal signaling. In plants, SEC- or SPY-mediated *O*-glycosylation modulates diverse pathways, such as cytokinin responses via TCP14/15 (Steiner et al. 2012), gibberellin signaling through DELLA protein REPRESSOR OF *ga1-3* (RGA) (Zentella et al. 2016), ethylene signaling transduction via EIN2 (Xu et al. 2023b), and auxin responses through TFs ARF6/8 (Wang et al. 2025). These studies collectively highlight *O*-glycosylation as a key regulatory node linking hormone signaling to transcriptional control. In this context, our work identifies the master ripening regulator SlNOR as a previously unrecognized nucleocytoplasmic *O*-glycosylation substrate and establishes that SlSEC1/SlSPY-mediated modifications of SlNOR directly regulate ethylene biosynthesis to control fruit ripening (Figs. 1–7; Figure S1 to S13).

Proteins containing TPR domains are recurrently implicated in ethylene regulation (Yoshida et al. 2005; Lin et al. 2008). For instance, ETHYLENE-OVERPRODUCER1 ETO1 interacts with ACS5 to tune ethylene biosynthesis in Arabidopsis (Yoshida et al. 2005), and SlTPR1 binds ethylene receptors Never ripe and ethylene resistant 1 (LeETR1) to modulate ethylene responses (Lin et al. 2008). Both SlSEC1 and SlSPY contain typical TPR domains (Fig. 1b and 1d), and our genetic evidence demonstrates that they function as positive regulators of ripening (Figs. 1 and 2; Figure S5).

Unlike the embryo-lethal *sec spy* double mutant in Arabidopsis (Hartweck et al. 2002), tomato *Slsec1 Slspy* double mutants are viable in this study (Fig. 1; Figure S6). This difference is explained by the presence of an additional SEC paralog in tomato, SlSEC2, which is absent in Arabidopsis. Public RNA-seq data show that SlSEC2 and SlSPY are highly expressed in seed tissues during early embryogenesis, whereas SlSEC1 expression is low (Figure S2c; Pattison et al. 2015). Consistently, *Slsec1-1* and *Slspy* single mutants exhibit partial germination and reproductive defects, while the *Slsec1-1 Slspy* double mutant shows more severe impairment yet remains capable of germination and pollen formation (Figure S6). These observations suggest that residual OGT activity, likely provided by SlSEC2, partially compensates for the loss of SlSEC1 and SlSPY during embryogenesis. Thus, the presence of SlSEC2 in tomato buffers against embryonic lethality, enabling viable double mutants and allowing direct study of SEC/SPY postembryonic functions in fruit ripening–a unique advantage of tomato as a model system.

Crosstalk often occurs between the pathways of SEC-mediated *O*-GlcNAcylation and SPY-mediated *O*-fucosylation (Aizezi et al. 2025). In Arabidopsis, AtSEC and AtSPY have been reported to function antagonistically—most prominently in gibberellin signaling and cytokinin signaling in plant development (Steiner et al. 2012; Zentella et al. 2017). However, in style development, AtSEC and AtSPY act synergistically, and both *O*-fucosylation and *O*-GlcNAcylation promote SPT (basic helix–loop–helix TF) binding to *PINOID* promoter to activate its transcription (Jiang et al. 2024). A cooperative role for SlSEC1 and SlSPY in promoting tomato fruit ripening was observed in our study. Pharmacological inhibition of either enzyme delayed the onset of ripening (Figures S1). Genetic disruption of SlSEC1 or SlSPY resulted in similar ripening delays, while the *Slsec1*-1 *Slspy* double mutant exhibited a more severe phenotype, including delayed breaker transition, reduced ethylene production, impaired softening, poor germination, retarded vegetative growth, and broad metabolic reprogramming (Fig. 1e–h;  Figures S2–S6), further supporting their synergistic action.

Despite their overall synergy, functional distinctions exist. Notably, SlSPY operates at multiple levels within the ethylene pathway. Xu et al. (2023b) demonstrated that SlSPY promotes ethylene signaling via *O*-fucosylation of SlEIN2. Our study reveals that SlSPY also influences ethylene biosynthesis through *O*-fucosylation and stabilization of SlNOR, a NAC TF that activates ethylene biosynthetic genes. Thus, SlSPY coordinately regulates both ethylene synthesis and perception–a dual-layer mechanism that may enable precise control of ripening. This dual-layer regulation may represent a general mechanism by which *O*-glycosyltransferases integrate hormonal pathways in plants. Consistent with this, the *Slspy* mutant exhibited greater insensitivity to exogenous ethylene than *Slsec1-1* in our postharvest assays (Fig. 2), indicating that SlSPY functions in both ethylene biosynthesis and signal transduction, whereas SlSEC1 appears to act primarily through ethylene biosynthesis via SlNOR regulation.

Molecular evidence further supports both the cooperative and differential roles of these enzymes. Substantial overlap exists between the SlSEC1 and SlSPY interactomes, including numerous nuclear and cytoplasmic proteins involved in cell wall remodeling, ethylene production, and volatile biosynthesis (Fig. 3a;  Supplementary Data Set S2). Both SlSEC1 and SlSPY physically interact with SlNOR (Fig. 3b), though with distinct subcellular patterns: SlNOR interacts with SlSPY predominantly in the nucleus, while its interaction with SlSEC1 occurs in the cytoplasm (Fig. 3d;  Figure S7). These distinct interaction patterns may underlie their differential contributions to ethylene response and ripening. Notably, 28.3% of the overlapping interactors are predicted to localize in chloroplasts (Figure S8b). However, our localization assays detected SlSEC1 and SlSPY predominantly in the nucleus and cytoplasm (Figure S7), and DeepLoc 2.1 prediction similarly indicates very low plastid localization probability (Table S2). A possible explanation is that plastid proteins are synthesized in the cytosol as precursor proteins and imported post-translationally, allowing SlSEC1 and SlSPY to modify them prior to import. Supporting this, Liang et al. (2021) demonstrated that SPY-mediated *O*-fucosylation of the chloroplast protein CPN20 occurs in the cytosol and negatively regulates its import. Consistent with this interpretation, recent glycoproteomic studies across plant species have likewise reported plastid-localized proteins among SEC/SPY targets (Bi et al. 2023; Li et al. 2023; Shrestha et al. 2024).

NOR is a NAC family TF whose N-terminal NAC domain mediates DNA binding, while the C-terminal TRR facilitates protein–protein interactions (Ooka et al. 2003; Ernst et al. 2004). The NAC domain is organized into 5 conserved subdomains (A–E) that form the DNA-binding interface. Structural studies indicate that subtle alterations within this domain can profoundly impair DNA-binding activity (Tran et al. 2004; Olsen et al. 2005; Harrington et al. 2019; Chun et al. 2023). We identified 3 *O*-glycosylation sites—Thr93, Thr134, and Ser165—within subdomains C and E of the SlNOR NAC domain (Fig. 4). Simultaneously mutating these sites abolished SlNOR's DNA-binding ability in EMSAs without altering its overall secondary structure (Fig. 6c–e; Figure S12), indicating that these 3 *O*-glycosylation sites are essential for SlNOR's DNA interaction. Interestingly, domain mapping revealed that SlSEC1 and SlSPY interact with the C-terminal TRR of SlNOR, not the modified NAC domain (Figure S9). This suggests a putative modular regulatory mechanism: the TRR domain serves as a docking site for the *O*-glycosyltransferases, which in turn modify the DNA-binding NAC domain.

*O*-glycosylation influences multiple aspects of protein function, including stability, activity and localization (Aizezi et al. 2025). The stabilization of proteins such as AtTCP14, AtPRR5, SlEIN2, and SlC3H39 has been associated with their *O*-glycosylation status, underscoring a conserved role for *O*-glycosylation in safeguarding key regulators (Steiner et al. 2016; Wang et al. 2020; Xu et al. 2023a, 2023b). Similarly, our data show that *O*-glycosylation does not alter SlNOR's nuclear localization but significantly enhances its accumulation in the nucleus (Fig. 5a, b, g). This effect stems from protection against both proteasome-dependent and -independent degradation, as demonstrated by in vitro degradation and in vivo CHX chase assays (Fig. 5c–f).

Beyond stability, *O*-glycosylation enhances transcriptional activity of TFs such as SPT and ARF6/8 (Jiang et al. 2024; Wang et al. 2025). In our study, the glycosylation-deficient mutant SlNOR^S/T-A^ showed severely compromised activation of the *SlACS2* and *SlACO1* promoters in dual-luciferase assays, even after normalization for protein abundance (Fig. 6a, b). This indicates that the activity loss is not solely due to reduced protein levels. Notably, neither SlSEC1 nor SlSPY alone activated these promoters, nor did their coexpression with SlNOR significantly enhance SlNOR-dependent activation in transient assays, indicating that their physical interaction does not boost SlNOR's transcriptional activity (Figure S11). In contrast to the inhibitory effect of Met138 oxidation on SlNOR's DNA-binding activity (Jiang et al. 2020), our in vitro glycosylation reconstitution experiments demonstrate that *O*-GlcNAcylation and *O*-fucosylation markedly enhance it in a concentration-dependent manner (Fig. 7; Figure S13). This opposing regulation by distinct PTMs implies how SlNOR integrates multiple modifications to fine-tune its transcriptional output.

**Figure 8:**
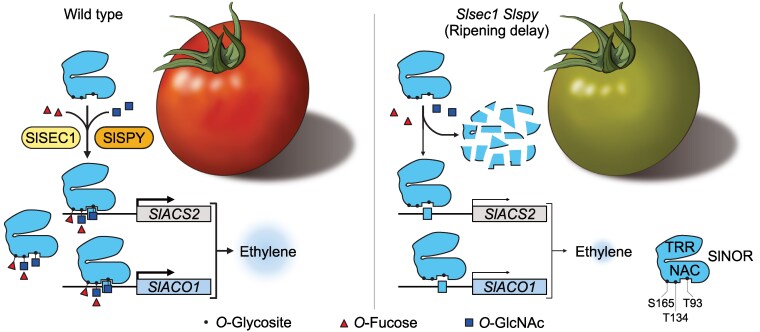
A proposed model for the *O*-glycosylation-mediated regulation of SlNOR in tomato ripening. SlNOR protein is composed of a NAC domain and a TRR domain. In WT fruit, the *O*-glycosyltransferases SlSEC1 and SlSPY physically interact with the TRR domain of SlNOR and catalyze *O*-GlcNAcylation and *O*-fucosylation at specific Ser/Thr residues (Thr93, Thr134, and Ser165, small dot) within the NAC domain. These *O*-GlcNAc (squares) and *O*-fucose (triangles) modifications promote SlNOR protein stability and enhance its DNA-binding affinity. This leads to effective transcriptional activation of ethylene biosynthesis genes, including *SlACS2* and *SlACO1*, during fruit ripening. In the *Slsec1 Slspy* double mutant, impaired SlNOR glycosylation reduces its stability and activity, diminishing the expression of ethylene-related genes and causing a significant delay in fruit ripening.

Taken together, our study establishes the role of *O*-glycosylation in orchestrating fruit ripening. A potential model was proposed to illustrate the role of SlSEC1- and SlSPY-mediated *O*-glycosylation for the post-translational control of SlNOR in fruit ripening (Fig. 8). In this model, the *O*-glycosyltransferases SlSEC1 and SlSPY act as critical upstream regulators of the master TF SlNOR. Upon physical interaction with the TRR domain, they catalyze the *O*-glycosylation of SlNOR at specific sites (Thr93, Thr134, and Ser165) within its NAC domain. These modifications confer enhanced protein stability and increase DNA-binding affinity, which synergistically boost SlNOR's ability to activate the transcription of key ethylene biosynthesis genes, *SlACS2* and *SlACO1*. This transcriptional amplification triggers ethylene biosynthesis and drives fruit ripening. Consequently, genetic ablation of both enzymes in the *Slsec1*-1 *Slspy* mutant disrupts this essential PTM, leading to diminished SlNOR stability and transcriptional activity on the *SlACS2* and *SlACO1* promoters, which manifests as reduced ethylene production and a delayed ripening phenotype.

While our study establishes a key mechanism, several exciting avenues remain. First, dissecting the individual contributions of the 3 glycosylation sites, particularly the dually modified Thr134 residue, will be crucial to understanding the specific roles of *O*-GlcNAc versus *O*-fucose. Second, NAC TFs often form homodimers or heterodimers (Olsen et al. 2005), investigating whether *O*-glycosylation influences SlNOR dimerization or its interaction with other ripening-related TFs is a key next step. Third, our interactome analysis identified numerous other promising ripening-related proteins as potential SlSEC1/SlSPY interactors (Fig. 3a;  Supplementary Data Set S2). Systematically validating these texture/volatile/hormone-linked candidates could reveal a broader role for *O*-glycosylation in coordinating diverse aspects of ripening. Finally, our findings have clear translational potential. Rather than employing blunt instruments like knocking out master regulators, modulating SlSEC/SlSPY activity or engineering specific glycosylation sites offers a precise molecular toolkit to fine-tune ripening, potentially extending shelf life and improving fruit quality in tomato and other horticultural crops.

## Material and methods

### Plant materials and growth conditions

WT tomato (*S. lycopersicum* L. cv. Ailsa Craig) and the generated mutant lines (*Slsec1-*1, *Slsec1-*2, *Slspy*, and *Slsec1-*1 *Slspy*) were grown in a greenhouse under a 16 h light (25 °C)/8 h dark (20 °C) photoperiod at 70% relative humidity. Flowers were tagged at anthesis to track fruit DPA and fruits were tagged at Br stage to track fruit developmental stages. Fruits were harvested at Br, and then at 3, 8 days after the Br stage (Br + 3 and Br + 8) stage and immediately frozen in liquid nitrogen and stored at −80 °C. *N. benthamiana* plants, used for transient expression assays, were grown under identical conditions.

### Constructs and plant transformation

To generate the *Slsec1-*1 and *Slspy* mutants, 2 single-guide RNAs (sgRNAs) targeting *SlSEC1* and *SlSPY* were designed using CRISPR-P (http://crispr.hzau.edu.cn/cgi-bin/CRISPR2/CRISPR). The sgRNA expression cassettes were cloned into the pYLCRISPR/Cas9pUbi-H binary vector via the Golden Gate method. The resulting constructs were sequence-verified and transformed into Ailsa Craig tomato via *Agrobacterium tumefaciens*-mediated cotyledon transformation as described by Ma et al. (2015). The *Slsec1-*1 *Slspy* double mutant was generated by crossing the homozygous single mutants. All primers used are listed in Supplementary Data Set S4.

### VIGS assays

VIGS assays were performed using the Tobacco rattle virus (TRV)-based vector system (Fu et al. 2005; Gao et al. 2018). A 300-bp fragment of phytoene desaturase gene (*PDS*), *SlSEC1*, or *SlSEC2*, was amplified from tomato cDNA and cloned into the pTRV2 vector to generate recombinant constructs pTRV2-PDS, pTRV2-SlSEC1, and pTRV2-SlSEC2, respectively. *A. tumefaciens* strain GV3101 carrying pTRV1 and the respective pTRV2 construct were coinfiltrated into IMG tomato fruits on one side. All primers used are listed in Supplementary Data Set S4.

### RT-qPCR

Total RNA was extracted from tomato fruit using the CTAB method. First-strand cDNA was synthesized using HiScript III RT SuperMix for qPCR (+gDNA wiper) (Vazyme, China). RT**-**qPCR was performed using ChamQ Universal SYBR qPCR Master Mix (Vazyme) on a Bio-Rad CFX96 Real-Time System (Bio-Rad, USA). Relative expression was calculated using the 2^−ΔΔCT^ method with *SlACTIN* as the internal reference. Three biological replicates were analyzed for each sample. Gene-specific primers are listed in Supplementary Data Set S4.

### Phenotype analysis

Fruit firmness was measured on the pericarp using a TA. XT.Plus texture analyzer (Stable Micro Systems, UK). Plant height was measured at 30 DPA. For seed germination, 25 seeds per genotype were incubated in distilled water at 25 °C with shaking (150 rpm), and germination status was recorded after 4 days. Pollen from individual flowers was stained with Alexander's stain and imaged under a Leica DM1000 LED microscope.

### Ethylene measurement

Ethylene production was measured by sealing fruits in a 300 mL gas-tight container for 2 h at 25 °C. A 1 mL headspace gas sample was withdrawn and analyzed using a 7890B gas chromatograph system (Agilent Technologies, USA).

### Chemical treatments

Inhibitor injection was performed as described in Zhao et al. (2025) with minor modifications. IMG stage fruits were injected into the central columella with 50 μL of 0.5 mM OSMI-4 (MCE, HY-114361, USA) as SEC inhibitor (Martin et al. 2018), 0.5 mM TMC647055 choline salt (MCE, HY-15591A, USA) as SPY inhibitor (Aizezi et al. 2024), or a mock solution (0.1% [v/v] Tween-20). All solutions were prepared in 0.1% Tween-20. For postharvest gas treatment, MG stage fruits were placed in sealed containers and treated with 100 ppm ethylene or 10 ppm 1-MCP at 25 °C. The gas environments were replenished every 24 h for 3 cycles, following a modified protocol (Li et al. 2020). For CHX chase assays, *N. benthamiana* leaves agroinfiltrated with expression constructs were subsequently injected with 5 mM CHX. Leaf samples were collected at indicated time points for immunoblot analysis with an anti-GFP antibody. Quantification of Western blot bands was measured using ImageJ software.

### Protein interaction assays

#### Yeast two-hybrid

Full-length *SlSPY*, *SlSEC1*, *SlNOR*, and truncated *SlNOR* (nSlNOR and cSlNOR) were cloned into the prey vector pGADT7 or bait vector pGBKT7. Double dropout medium (DDO) and quadruple dropout medium (QDO) were supplied by Coolaber in China. Yeast cultures were spotted in a 10-fold serial dilution series on DDO and QDO media. The plates were inverted and incubated at 30 °C for 3 d, after which they were photographed.

#### Bimolecular fluorescence complementation

BiFC assay was carried out according to our previous method (Cao et al. 2023). Full-length coding sequences were cloned into vectors containing the N-terminal (nYFP) or C-terminal (cYFP) fragments of YFP. *Agrobacterium* strains carrying the nYFP and cYFP constructs were coinfiltrated into *N. benthamiana* leaves. YFP fluorescence was imaged 48 h postinfiltration using an LSM 780 confocal microscope (Carl Zeiss, Germany).

#### Firefly LCI

Coding sequences were cloned into pCAMBIA1300 vectors containing the N-terminal (nLUC) or C-terminal (cLUC) fragments of luciferase. *Agrobacterium* strains were coinfiltrated into *N. benthamiana* leaves. Two days later, leaves were infiltrated with 0.2 mM D-luciferin, and luminescence was imaged using a NightSHADE LB 985 system (Berthold Technologies, Germany).

All primers used are listed in Supplementary Data Set S4.

### Subcellular localization

Subcellular localization was performed as previously described (Xu et al. 2023b). Full-length coding sequences of *SlSEC1* and *SlSPY* were cloned into the pCAMBIA2300-eGFP vector. Constructs were transformed into *A. tumefaciens* strain GV3101 and transiently expressed in *N. benthamiana* leaves. SYP122 was used as a plasma membrane marker. GFP signal was observed 48 h postinfiltration using an LSM 780 confocal microscope (Carl Zeiss, Germany). All primers used are listed in Supplementary Data Set S4.

### Protein immunoblot analyses

Total proteins were extracted using an extraction buffer [100 mM Tris-HCl (pH 7.5), 100 mM KCl, 10% (v/v) glycerol, 2 mM DTT, 1 mM PMSF, 1% (v/v) Triton X-100, and 1× protease inhibitor cocktail]. Nuclear proteins of leaves at 60 days postgermination were extracted using a commercial Plant Nucleoprotein Extraction Kit (Solarbio, #EX2051, USA). Proteins were resolved by SDS-PAGE and transferred to PVDF membranes. Membranes were blocked with 5% nonfat milk (for standard antibodies) or 3% BSA (for CTD110.6 and AAL) and probed with primary antibodies: rabbit anti-NOR polyclonal antibody (Abmart, China; 1:500), mouse anti-His monoclonal antibody (EASYBIO, BE2019, China; 1:5,000), rabbit anti-GFP polyclonal antibody (EASYBIO, BE7010, China; 1:5,000), mouse anti-*O*-GlcNAc monoclonal antibody CTD110.6 (Cell Signaling Technology, #9875S, USA; 1:2,000), or biotinylated AAL (Vector Labs, B-1395, China; 1:10,000). Signals were visualized with HRP-conjugated secondary antibodies and chemiluminescence (ECL). Ponceau S or Coomassie Brilliant Blue staining served as a loading control.

### Recombinant protein expression and purification

Protein expression was carried out as previously described (Cao et al. 2023). Coding sequences for the full-length SlNOR protein and truncated SlSEC1/SlSPY were cloned into the pET32a vector, while the TPR domain of SlSPY was cloned into the pGEX4T-1 vector. Constructs were transformed into *Escherichia coli* Transetta (DE3) cells (TransGen Biotech, China). Primers used for vector construction are listed in Supplementary Data Set S4. Protein expression was induced with 0.5 mM isopropyl β-D-1-thiogalactopyranoside at 16 °C for 20 h. Recombinant proteins were purified using His-tag or GST-tag affinity chromatography. His-tagged proteins were purified using a ProteinIso Ni-NTA gravity column (TransGen Biotech, China) and GST-tagged proteins were purified using a Protein Purification Kit (Beyotime, China).

### Cell-free degradation assay

Cell-free degradation assay was performed as previously described (Wang et al. 2009). The WT or *Slsec1-*1 *Slspy* mutant fruits were ground in liquid nitrogen. SlNOR-His (50 ng) was incubated with 100 μL of total protein extracts from WT or *Slsec1-*1 *Slspy* mutant fruits, with or without the proteasome inhibitor MG132 (50 μM). The protein blots were analyzed using an anti-His antibody. Quantification of Western blot bands was measured using ImageJ software.

### CD spectroscopy

The CD measurements were recorded on a J-1500 (JASCO) from a quartz cuvette with an optical path of 1 mm. SlNOR-His and SlNOR^S/T-A^-His were purified under native conditions. Bacterial pellets were resuspended in lysis buffer (50 mM Tris-HCl, 300 mM NaCl, 10 mM imidazole, 1 mM DTT, 5% glycerol) and lysed by gentle sonication on ice. Clarified lysates were incubated with Ni-NTA resin equilibrated in the same buffer. After washing with native wash buffer (50 mM Tris-HCl, 300 mM NaCl, 60 mM imidazole, 1 mM DTT, 5% glycerol), bound proteins were eluted with native elution buffer (50 mM Tris-HCl, 300 mM NaCl, 250 mM imidazole, 1 mM DTT, 5% glycerol). Eluted proteins were desalted using PD-10 columns (GE Healthcare, UK) and buffer-exchanged into 10 mM Tris-HCl buffer (pH 7.4) at a final concentration of 0.2 mg/mL for CD analysis. All steps were performed at 4 °C. Three technical replicates were analyzed for each sample, and the mean value is used for CD spectra.

### Dual-luciferase assay

The dual-luciferase reporter assay was as described in Gao et al. (2020). The coding sequence of SlNOR or SlNOR^S/T-A^ was cloned into the 35S promoter-driven pEAQ-HT-GFP vector (effector). The promoter regions (1 to 2 kb) of *SlACS2* and *SlACO1* were cloned into the pGreenII 0800-LUC vector (reporter). *Agrobacterium* strains carrying the Effector and reporter constructs were coinfiltrated into *N. benthamiana* leaves. Firefly (LUC) and Renilla (REN) luciferase activities were measured 48 h later using the Dual-Luciferase Reporter Assay System (Promega, USA) and a GloMax 96 microplate reader. The LUC/REN ratio was calculated to represent transcriptional activity. At least 4 biological replicates were performed for each combination. All primers used are listed in Supplementary Data Set S4.

### In vitro *O*-glycosylation assay

In vitro glycosylation was performed as previously described (Jiang et al. 2024). For *O*-GlcNAcylation, 10 μg of SlNOR-His was incubated with 1 μg of cSlSEC1-His (360 to 964 aa and 5TPR plus catalytic domain) in a 100 μL reaction containing 20 mM Tris-HCl (pH 7.2), 12.5 mM MgCl_2_ and 200 μM UDP-GlcNAc (MCE, HY-148596). For *O*-fucosylation, 10 μg of SlNOR-His was incubated with 1 μg of cSlSPY-His (360 to 850 aa, 3TPR plus catalytic domain) in a 100 μL reaction containing 50 mM Tris-HCl (pH 8.2), 50 mM NaCl, 5 mM MgCl_2_ and 200 μM GDP-fucose (MCE, HY-134433). Reactions were incubated for 2 h at 25 °C, and the products were used in EMSA experiments.

### Electrophoretic mobility shift assay

EMSA was performed using the LightShift^®^ Chemiluminescent EMSA Kit (Thermo Fisher Scientific, USA) according to our previous method (Cao et al. 2023). Biotin-labeled dsDNA probes corresponding to the *SlACS2* and *SlACO1* promoters were incubated with glycosylated or nonglycosylated recombinant SlNOR-His protein. Reaction products were separated on a 5% native polyacrylamide gel in 0.5× Tris-borate-EDTA buffer at 150 V for 40 min and visualized following the kit instructions.

### GST pull-down and MS

Recombinant GST-nSlSPY (38 to 452 aa and 1 to 11 TPR), GST-nSlSEC1 (1 to 502 aa and 1 to 13 TPR) or GST alone (control) was immobilized on glutathione-sepharose beads and purified according to the GST-tag Protein Purification Kit (Beyotime, P2262, China). The beads were incubated overnight at 4 °C with total protein extracts from tomato fruit. After extensive washing, bound proteins were eluted and sent for identification on an Orbitrap Exploris 480 MS platform at PTM Biolabs (Hangzhou, China).

#### Chromatographic conditions

The mobile phase consisted of solvent A (0.1% formic acid and 2% acetonitrile/in water) and solvent B (0.1% formic acid and 90% acetonitrile/in water). Tryptic peptides were separated with the following gradient: 0 to 22 min, 6%-35% B; 22 to 26 min, 35% to 80% B; 26-30 min, 80% B, and all at a constant flow rate of 550 nl/min on an EASY-nLC 1,200 UPLC system (ThermoFisher, USA). MS conditions: The electrospray voltage applied was 2,300 V. Precursors and fragments were analyzed at the Orbitrap detector. The full MS scan resolution was set to 60,000 for a scan range of 350 to 1,800 *m/z*. The MS/MS scan resolution was set to 15,000. Up to 20 most abundant precursors were then selected for further MS/MS analyses with 20 s dynamic exclusion. The higher-energy collisional dissociation (HCD) fragmentation was performed at a normalized collision energy of 28%. Automatic gain control (AGC) target was set at 50%, with an intensity threshold of 5,000 ions/s and a maximum injection time of 200 ms. The resulting MS/MS data were processed using PD search engine (v2.4). Raw MS files were searched against Blast_Solanum_lycopersicum_4081_PR_20241023.fasta (34,662 entries, fasta. file attached in the Data Availability part) database. Trypsin/P was specified as cleavage enzyme allowing up to 2 missing cleavages. The mass tolerance for precursor ions was set as 10 ppm in first search, and the mass tolerance for fragment ions was set as 0.02 Da. Carbamidomethyl on Cys was specified as a fixed modification, and acetylation on protein N-terminal, oxidation on Met were specified as variable modifications. Peptide scoring is required to be higher than 20, and peptide confidence is set to High for identification results.

### IP-MS for *O*-glycosylation site identification

IP-MS analysis was performed using a modified procedure described by Xu et al. (2023b). SlNOR-Flag was transiently expressed in *N. benthamiana* leaves. Total protein was extracted, and the lysate was incubated with anti-Flag magnetic beads (ABclonal, AE037, China) overnight at 4 °C. Beads were washed, and immunoprecipitated proteins were eluted and sent for *O*-glycosylation site identification on an Orbitrap Fusion Lumos MS platform at Qinglian Biotech (Beijing, China) with EThcD fragmentation.

#### Chromatographic conditions

The mobile phase consisted of solvent A (100% water, 0.1% formic acid) and solvent B (80% acetonitrile, 0.1% formic acid). Tryptic peptides were separated with the following gradient: 0 to 11 min, 7% to 15% B; 11 to 48 min, 15% to 25% B; 48 to 68 min, 25% to 40% B; 68 to 69 min, 40% to 100% B; 69 to 75 min, 100% B. MS conditions: The MS was operated in a data-dependent acquisition mode, with a full scan range of *m/z* 350 to 1550. The resolution for the primary MS was set to 60,000 (at 200 *m/z*), with an AGC target of 4 × 10^5^ and a maximum injection time of 50 ms for the C-trap. The top 40 most intense ions from the full scan were selected for fragmentation using HCD combined with electron-transfer dissociation, followed by secondary MS analysis. The secondary MS was conducted in “Top Speed” mode, with ions detected using an ion trap. The AGC was set to 5 × 10^4^, with a maximum injection time of 50 ms, and the collision energy for peptide fragmentation was set to 25%. Raw MS files were processed using pGlyco3 for glycopeptide identification (Zeng et al. 2021). Search was performed against the SlNOR.fasta with a stringent 1% false discovery rate at the glycopeptide-spectrum match level as mentioned in our previous study (Zhao et al. 2025).

Ion type assignments and relative intensities (%) of fragment ions from EThcD tandem mass spectra to identify *O*-glycosites of SlNOR are listed in Supplementary Data Set S3.

### Fruit metabolomics

The extraction, detection, and quantitative analysis of secondary metabolites in WT and mutant fruits at the Br + 8 stage were performed by Metware Biotechnology Co., Ltd. (Wuhan, China). Pearson correlation coefficients were calculated across all samples (Supplementary Data Set S1).

### Phylogenetic analysis

Tomato orthologs of SlSEC and SlSPY were identified by performing BLAST searches against plant genomes from *Arabidopsis thaliana* (At), *Oryza sativa* (Os), *Zea mays* (Zm), *Vitis vinifera* (Vv), *Populus trichocarpa* (Pt), *Petunia hybrida* (Ph), and *Ricinus communis* (Rc) in Phytozome 14 (https://phytozome-next.jgi.doe.gov/). Gene model numbers of each species are listed in Table S1. Multiple sequence alignment was performed using the MUSCLE program (Supplemental File S1). Phylogenetic trees were constructed with MEGA 7 using the neighbor-joining method with 1,000 bootstrap replicates (Supplemental File S2).

### Bioinformatics analysis

Genome-wide collinearity analysis was performed using MCScanX implemented in TBtools based on the tomato genome annotation ITAG4.0. The syntenic relationship between *SlSEC1* and *SlSEC2* was visualized using TBtools (Chen et al. 2023). Syntenic blocks were identified using default parameters, and collinear gene pairs were extracted from the MCScanX output (Supplemental File S3).

Protein sequences of SlSEC1, SlSPY, and their corresponding mutants (*Slsec1-*1, *Slsec1-*2, and *Slspy*) were obtained by translating nucleotide sequences using the ExPASy Translate tool (http://web.expasy.org/translate/). Domain prediction was performed using the NCBI Conserved Domain Database (https://www.ncbi.nlm.nih.gov/Structure/cdd/wrpsb.cgi) to identify the TPR and catalytic domains of SlSEC1 and SlSPY, as well as the N-terminal NAC domain and C-terminal TRR of SlNOR.

For proteomics data analysis, subcellular localization was predicted using WolF Psort (v0.2) (Horton et al. 2007). Functional annotation was carried out through GO and KEGG pathway mapping using the DAVID database (Sherman et al. 2022). Protein domains were further annotated with PfamScan (v1.6) against the Pfam-A.hmm library (v37.0) (https://www.ebi.ac.uk/interpro/entry/pfam/#table). Clusters of orthologous groups of proteins (KOG) classification was performed using eggNOG (v5.0.2, http://eggnog5.embl.de/#/app/home). Subcellular localization predictions of SlSEC1, SlSEC2, and SlSPY were obtained using the DeepLoc2.1 tool (Ødum et al. 2024; https://services.healthtech.dtu.dk/services/DeepLoc-2.1/).

### Statistical analysis

All experiments were conducted with at least 3 independent biological replicates, each replicate containing 4 fruits, unless otherwise specified. Values are means ± standard deviation (SD). Statistical significance was determined using Student's *t*-test or one-way ANOVA with Tukey's multiple test with SPSS software (v27). Differences were considered significant at *P* < 0.05. All statistical analysis data were shown in Supplementary Data Set S5.

### Gene accessions

The genes described in the study can be found in the SOL Genomics Network (http://solgenomics.net/) under the following accession numbers: *SlNOR* (Solyc10g006880), *SlSEC1* (Solyc09g097830), *SlSEC2* (Solyc05g053835), *SlSPY* (Solyc09g010180), *SlACS2* (Solyc01g095080), *SlACO1* (Solyc07g049530), and *SlActin* (Solyc03g078400).

## Supplementary Material

koag144_Supplementary_Data

## Data Availability

Datasets of GST pull-down interactome of both SlSEC1 and SlSPY are available at the ProteomeXchange Consortium via the iProX partner repository (Ma et al. 2019) with the identifier PXD072030. iProX accession: IPX0014723000 at https://www.iprox.cn/page/PSV023.html;?url=1765871792907EzyN with password 3I7p.
