# Supplementary material for: SlSEC1- and SlSPY-mediated O-glycosylation stabilizes the transcription factor SlNOR to promote tomato fruit ripening
Source: Plant Cell. 2026 May 14;38(6):koag144. doi: 10.1093/plcell/koag144 (PMC13274654; doi:10.1093/plcell/koag144)
Supplement: koag144_Supplementary_Data [file koag144_supplementary_data.zip › Supplementary Figure.pdf]

SlSEC1- and SlSPY-mediated *O*-glycosylation stabilizes the SlNOR transcription factor to promote tomato fruit ripening

Yu-Di Wu[1], Ruo-Han Ou[1], Can Yang[1], Tong-Hao Cui[1], Qian-Yu Wang[1], Yu-Yang Mei[1], Jia-Fei Qian[1], Yi-Long Liu[1], Yuan-Jiang Pan[2], Zhi-Ping Deng[3], Qing-Qiu Gong[4], Jing Li[5], Zhen-Yu Qi[6], Yan-Na Shi[1], Donald Grierson[1,7], Bo Zhang[1], Kun-Song Chen[1], Xian Li[1,*], Xiao-Yong Zhao[1,*]

[1]Zhejiang Key Laboratory of Horticultural Crop Quality Improvement, Zhejiang University, Hangzhou 310058, P.R. China

[2]Department of Chemistry, Zhejiang University, Hangzhou 310058, P.R. China

[3]Institute of Virology and Biotechnology, Zhejiang Academy of Agricultural Sciences, Hangzhou 310021, P.R. China

[4]School of Life Sciences and Biotechnology, Shanghai Jiao Tong University, Shanghai 200240, P.R. China

[5]College of Chemical and Biological Engineering, Zhejiang University, Hangzhou, 310058, P.R. China

[6]Agricultural Experiment Station of Zhejiang University, Zhejiang University, Hangzhou 310058, P.R. China

[7]Plant and Crop Sciences Division, School of Biosciences, University of Nottingham, Loughborough, LE12 5RD UK

*Author for correspondence: xiaoyongzhao@zju.edu.cn (X.Y.Z); xianli@zju.edu.cn (X.L.);

The author(s) responsible for distribution of materials integral to the findings presented in this article in accordance with the policy described in the Instructions for Authors (https://academic.oup.com/plcell/pages/General-Instructions) are: Xian Li (xianli@zju.edu.cn) and Xiao-Yong Zhao (xiaoyongzhao@zju.edu.cn).

Short title: *O*-Glycosylation of SlNOR in fruit ripening

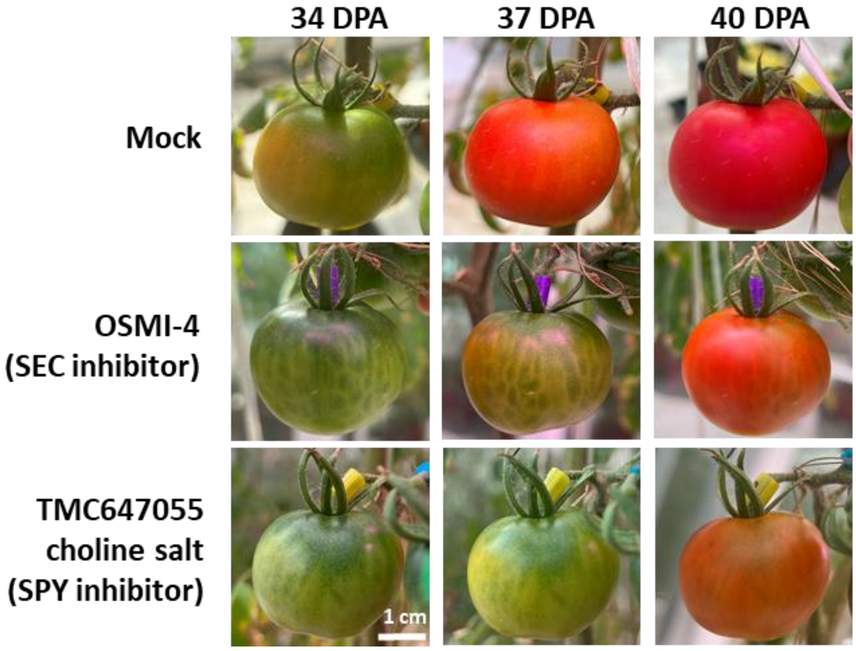

Supplementary Figure S1. Pharmacological inhibition of SlSEC- or SlSPY-mediated *O*-glycosylation delays fruit ripening (Supports Figure 1). Phenotypes of WT fruits injected with a mock solution (0.1% Tween-20), the SEC inhibitor OSMI-4 (0.5 mM), or the SPY inhibitor TMC647055 (0.5 mM) at the immature green (IMG) stage. Scale bar, 1 cm. DPA: days post-anthesis.

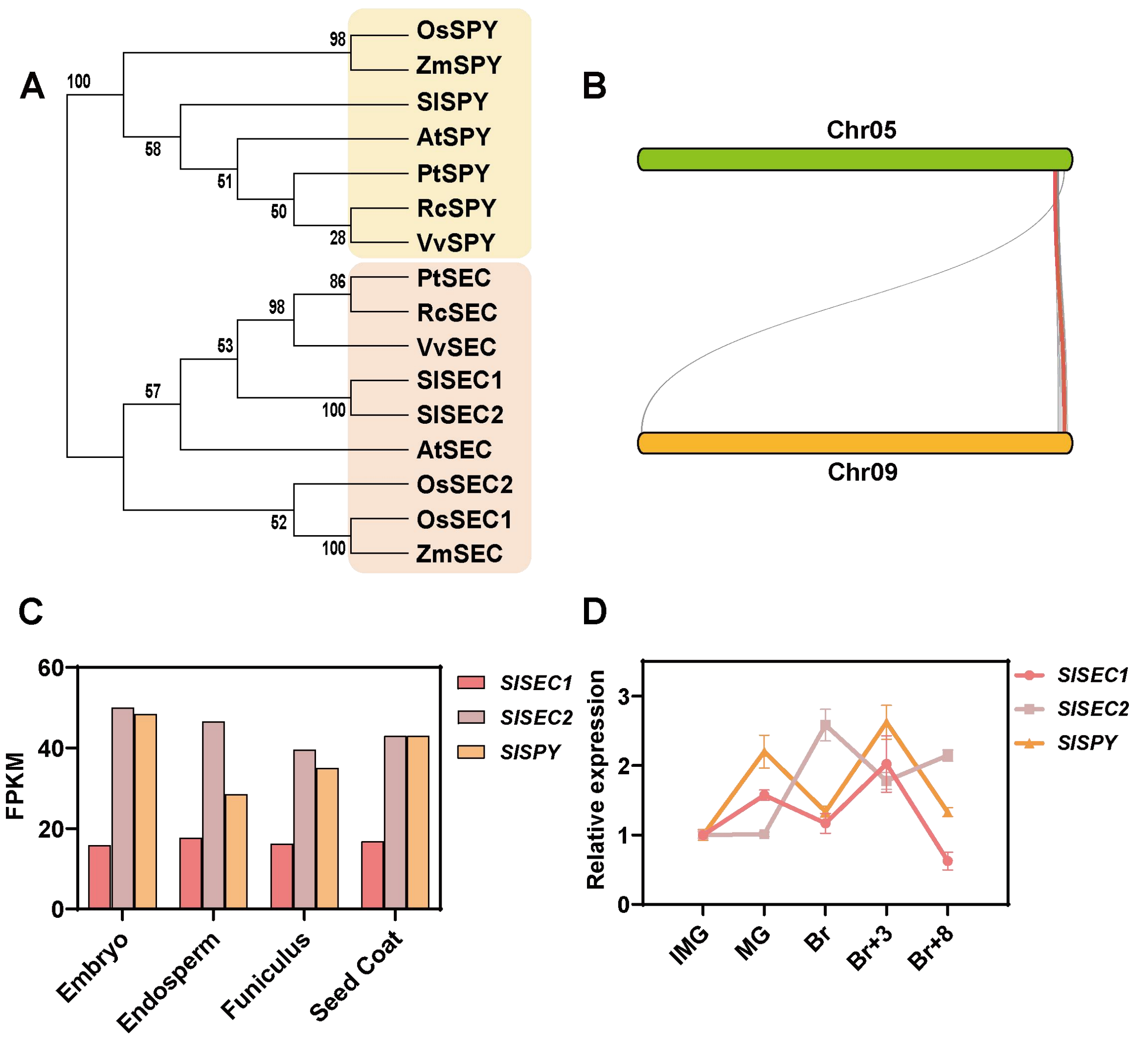

Supplementary Figure S2. Analysis of SlSEC and SlSPY gene families in tomato (Supports Figure 1). A) Phylogenetic analysis of SEC and SPY from representative plant species including *Arabidopsis thaliana* (At), *Solanum lycopersicum* (Sl), *Oryza sativa* (Os), *Zea mays* (Zm), *Vitis vinifera* (Vv), *Populus trichocarpa* (Pt) and *Ricinus communis* (Rc). The SPY clade (upper panel) and the SEC clade (lower panel) are clearly separated. Bootstrap values (%) from 1,000 replicates are shown at the nodes. **B)** Synteny relationship between the genomic regions containing *SlSEC1* and *SlSEC2* in the tomato genome. Grey curves represent syntenic gene pairs within the collinear block, and the positions of *SlSEC2* (on chromosome 05) and *SlSEC1* (on chromosome 09) are highlighted in red. **C)** Expression levels (FPKM) of *SlSEC1*, *SlSEC2* and *SlSPY* in seed tissues during early seed development. Transcript abundance in embryo, endosperm, funiculus and seed coat at 4 days post anthesis (4 DPA). RNA-seq data were retrieved from the TomExpress platform (https://tomexpress.gbfwebtools.fr/query). **D)** RT-qPCR analysis of *SlSEC1*, *SlSEC2* and *SlSPY* transcript levels in tomato fruits at indicated stages, including IMG, MG, Br, Br+3, and Br+8. Values are mean ± SD (n = 3 independent biological replicates).

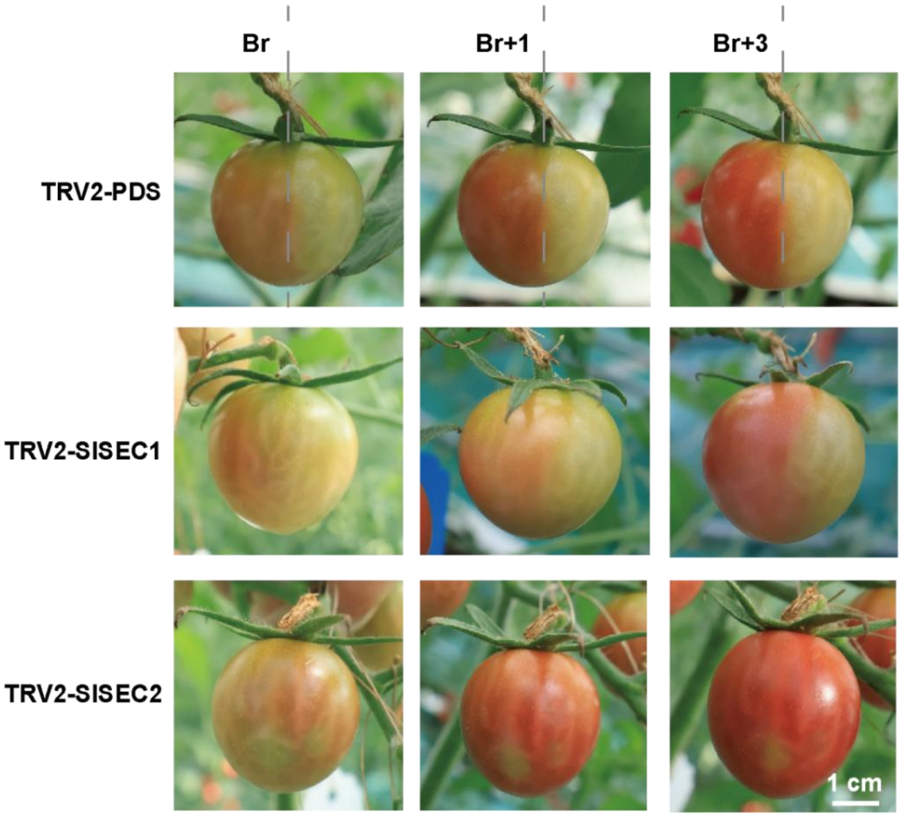

Supplementary Figure S3. Virus-induced gene silencing (VIGS) of SlSEC1 and SlSEC2 in tomato fruits (Supports Figure 1). TRV2-PDS (phytoene desaturase gene) is a positive control. Representative fruits at the Br, Br+1, and Br+3 stages are shown. Scale bar, 1 cm.

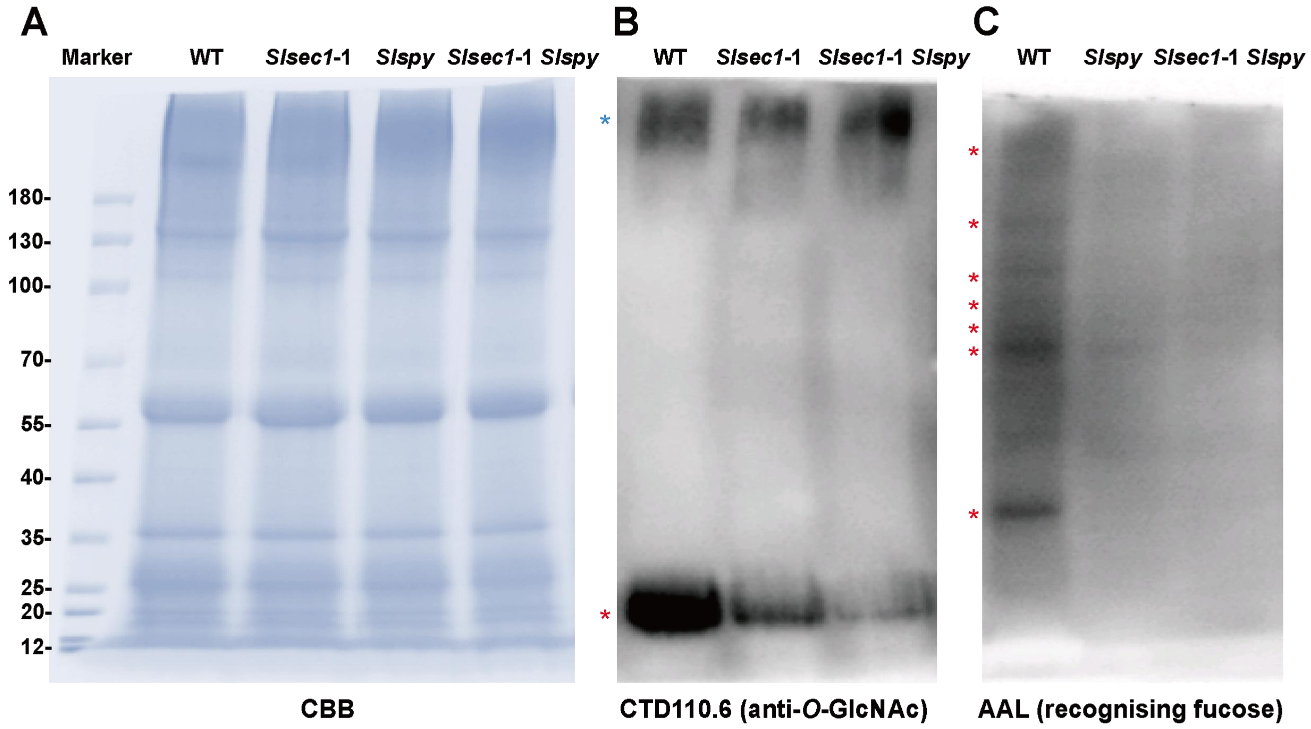

Supplementary Figure S4. Loss of SlSEC1 or SlSPY reduces global *O*-glycosylation levels in nuclear proteins (Supports Figure 1). A) Coomassie Brilliant Blue (CBB) staining of nuclear protein extracts from WT and mutant leaves at 60 days post germination (DPG), showing comparable protein loading. **B)** Immunoblot analysis of global *O*-GlcNAcylation levels in nuclear proteins using an anti-*O*-GlcNAc antibody CTD110.6. Signals are reduced in *Slsec1*-1 and *Slsec1*-1 *Slspy* mutants compared to WT. **C)** Lectin blot analysis of global *O*-fucosylation in nuclear proteins using biotinylated AAL. Signals are markedly reduced in *Slspy* and *Slsec1*-1 *Slspy* mutants. The red asterisk shows SlSEC1-specific *O*-GlcNAcylated/SlSPY-specific fucosylated protein bands and the blue asterisk shows non-specific signals in nuclear proteins in **(B)** and **(C)**

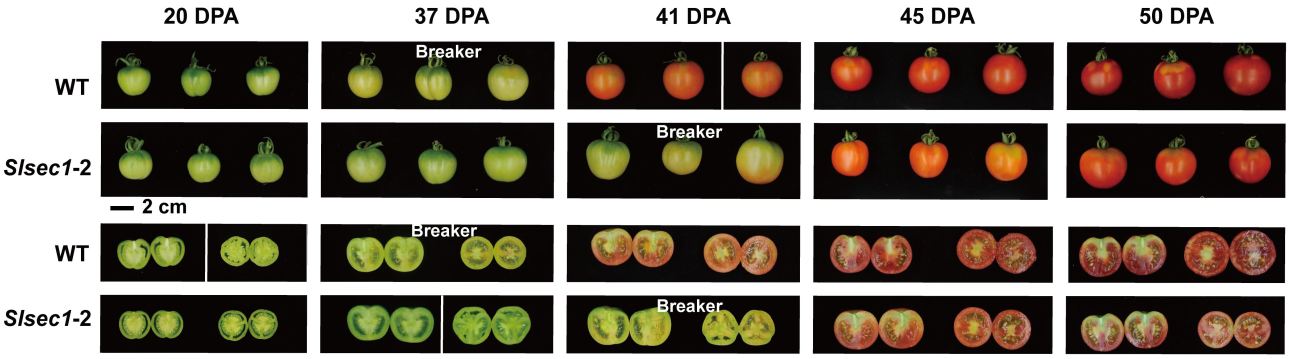

Supplementary Figure S5. The independent allele *Slsec1*-2 shows a delayed ripening phenotype (Supports Figure 1). Fruit ripening progression in WT and *Slsec1*-2 was shown at the indicated DPA. The Br stage is highlighted with a white box. Scale bar, 2 cm.

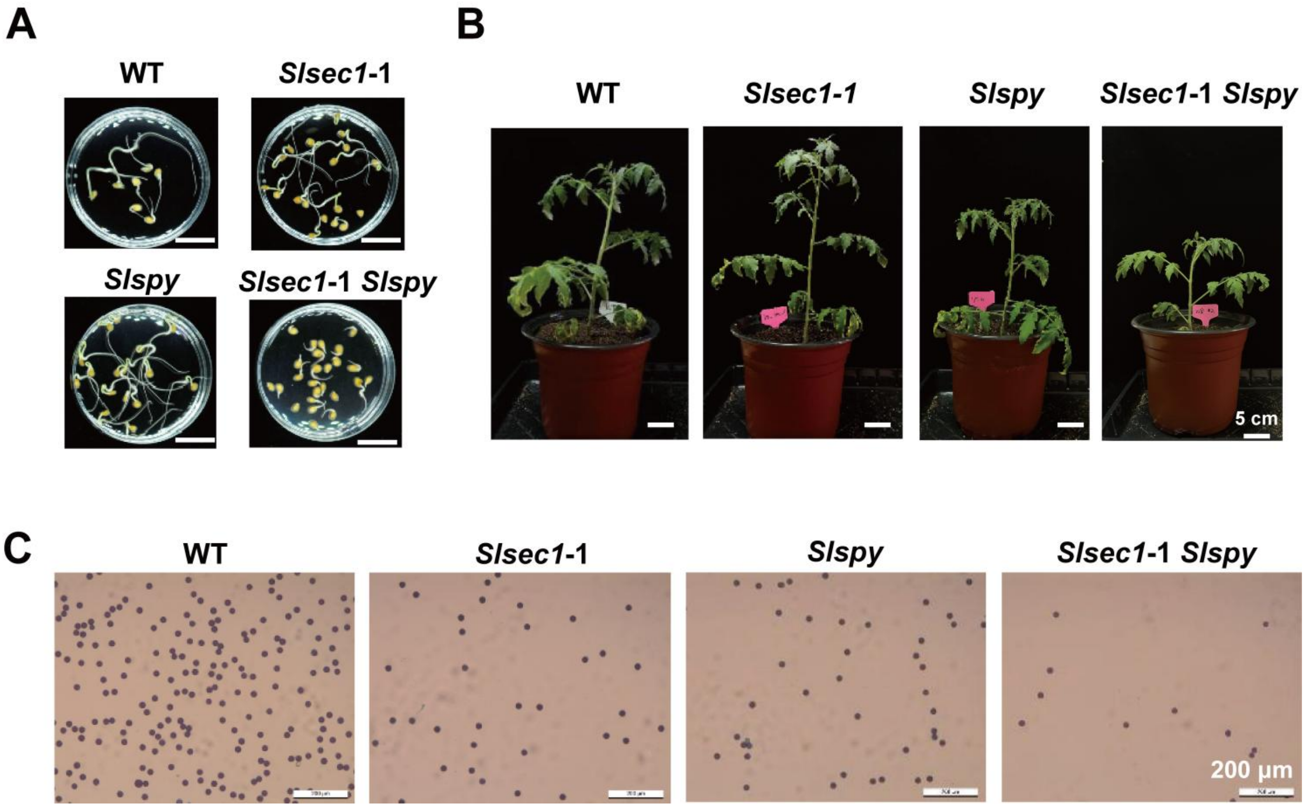

Supplementary Figure S6. Mutants *Slsec1*-1, *Slspy*, and *Slsec1*-1 *Slspy* display pleiotropic developmental defects (Supports Figure 1). A) Seed germination phenotypes of WT and mutant lines after 4 days of incubation. Scale bar, 2 cm. **B)** Vegetative growth phenotypes of 30 DPG in WT and mutant plants. Scale bar, 5 cm. **C)** Pollen density from WT and mutant flowers. Scale bar, 200 μm.

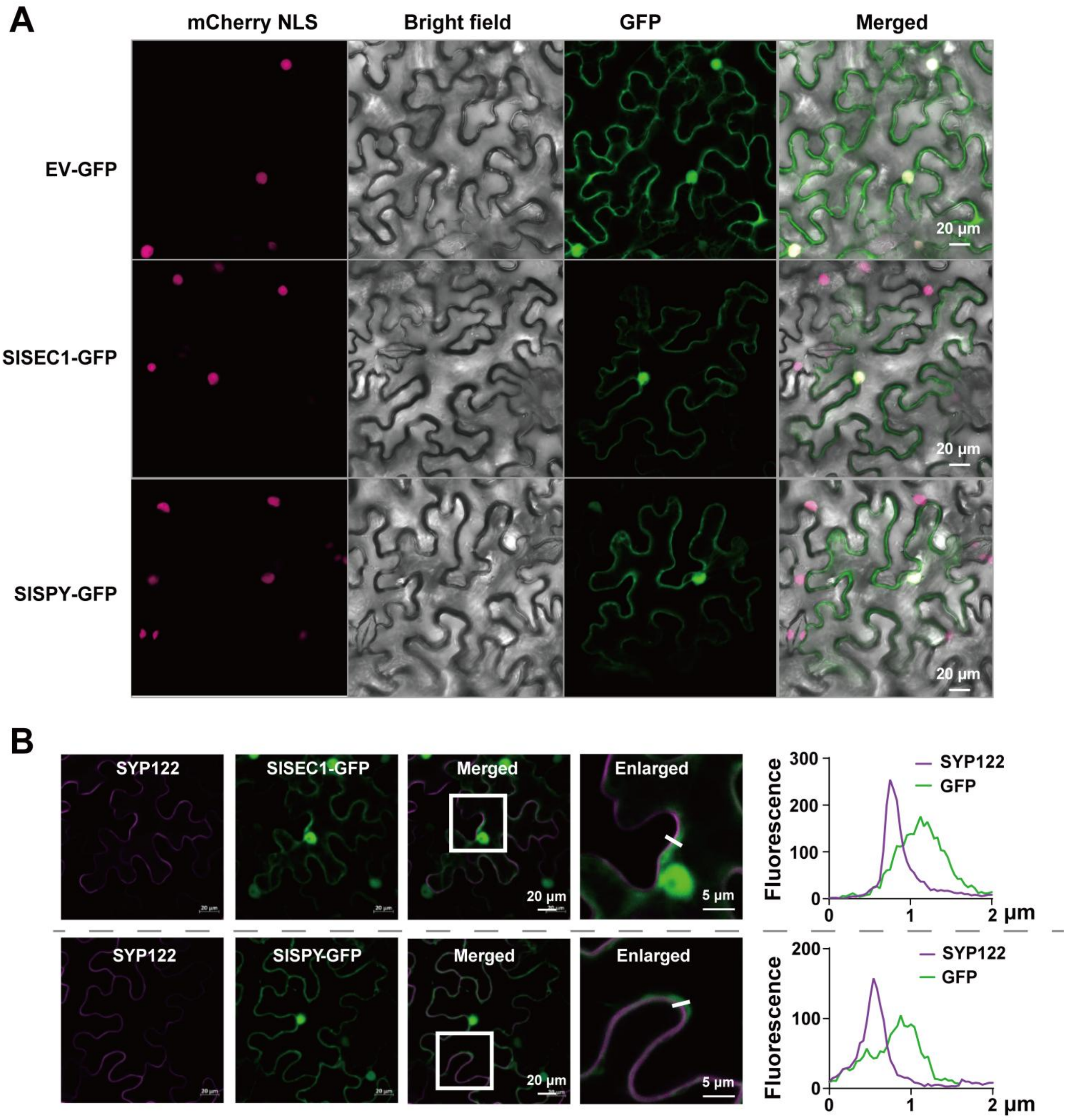

Supplementary Figure S7. SlSEC1 and SlSPY are localized in the nucleus and cytoplasm (Supports Figure 3). A) Confocal microscopy of *N. benthamiana* epidermal cells transiently expressing GFP, SlSEC1-GFP, or SlSPY-GFP. A nuclear mCherry fusion marks the nucleus. Empty vector (EV)-GFP is a control. Scale bars, 20 µm. **B)** Co-localization analysis of SlSEC1-GFP or SlSPY-GFP with the plasma membrane marker syntaxin of plant 122 (SYP122). Fluorescence intensity line-scan profiles show no overlap between GFP and SYP122signals, indicating that neither SlSEC1 nor SlSPY localizes to the plasma membrane. Scale bars, 20 µm (merged images) and 5 µm (enlarged views).

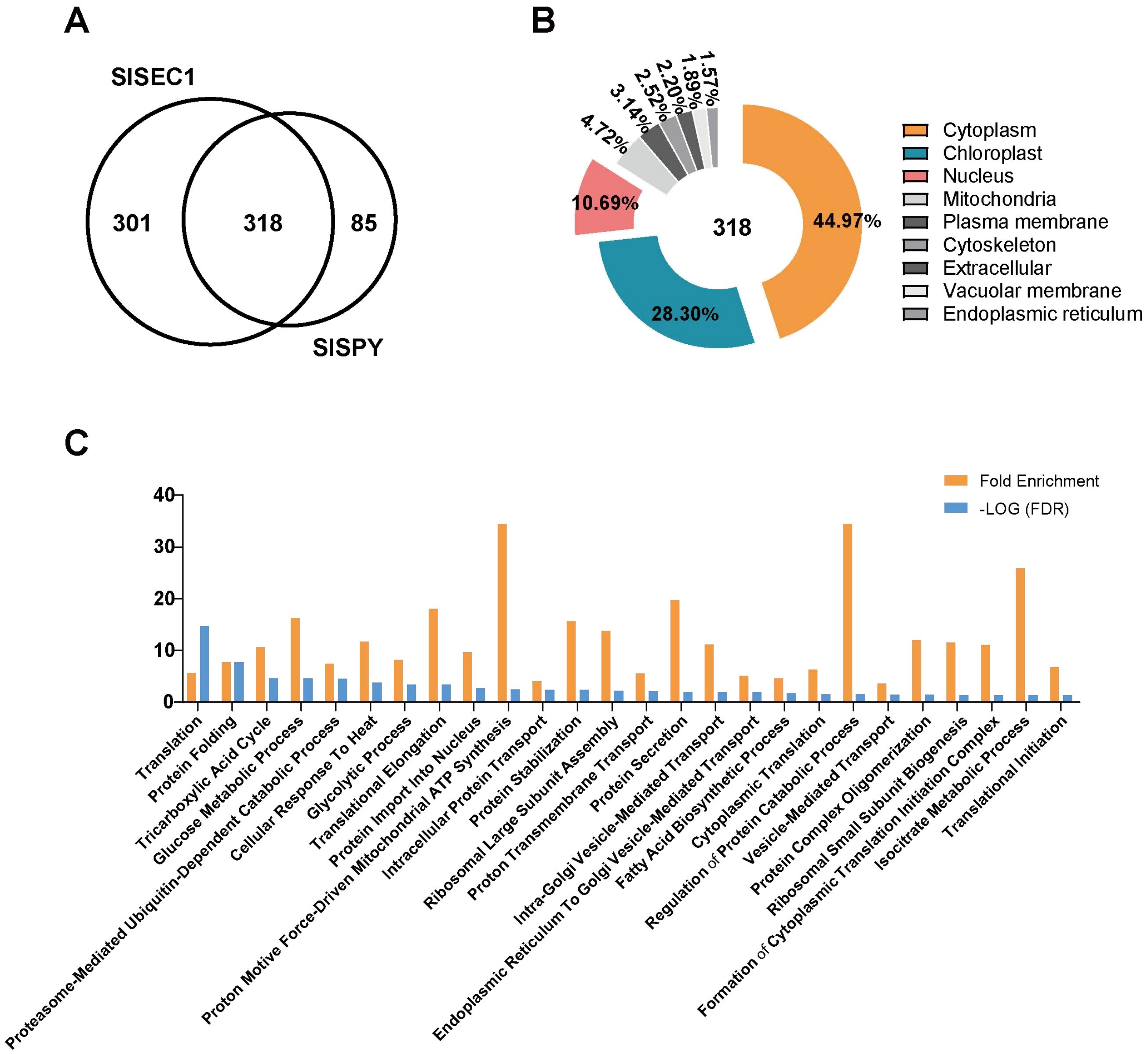

Supplementary Figure S8. Interactome analysis reveals overlapping interactors between SlSEC1 and SlSPY (Supports Figure 3). A) Venn diagram showing the overlap between potential SlSEC1 interactors (619 proteins) and SlSPY interactors (403 proteins) identified by GST pull-down MS. **B)** Predicted subcellular localization of the 318 overlapping interactors. **C)** Gene Ontology (GO) enrichment analysis of the molecular functions of the 318 overlapping interactors. Enriched terms with an FDR < 0.05 are shown.

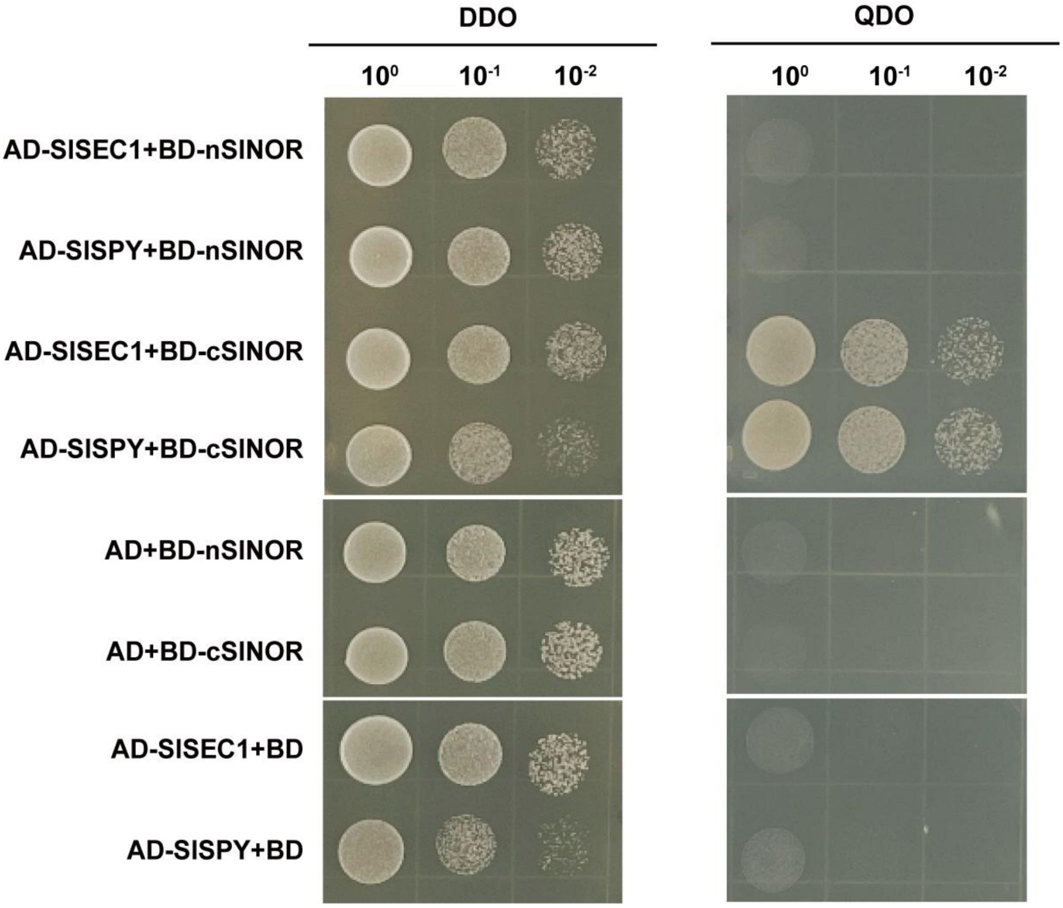

Supplementary Figure S9. The C-terminal TRR of SlNOR mediates its interaction with SlSEC1 and SlSPY (Supports Figure 3). Yeast two-hybrid (Y2H) assays testing interactions between SlSEC1/SlSPY (prey) and truncated domains of SlNOR (bait): the N-terminal NAC domain (nSlNOR, 1–180 aa) and the C-terminal TRR domain (cSlNOR, 181–355 aa). Yeast cultures were spotted in a 10-fold serial dilution series on non-selective (DDO) and selective (QDO) media. Growth on selective medium (QDO) indicates a positive interaction.

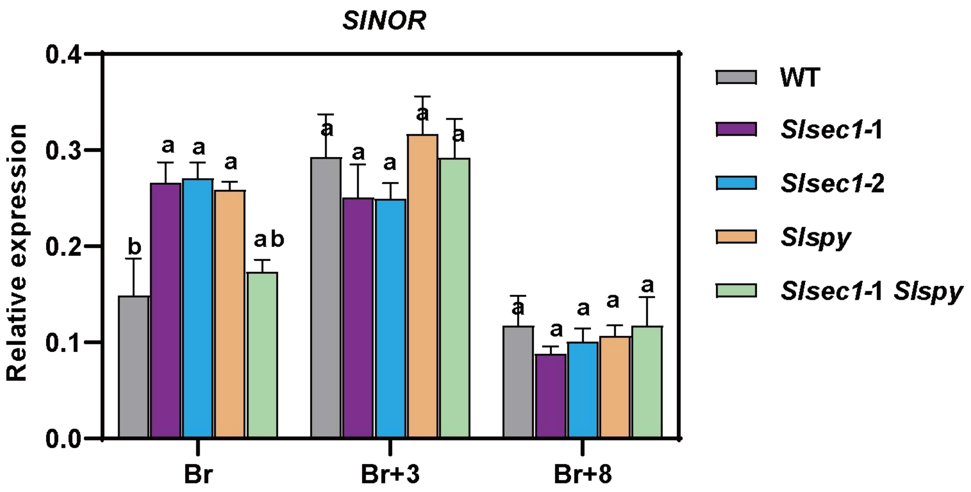

Supplementary Figure S10. RT-qPCR analysis of *SlNOR* expression in WT and mutant fruits (Supports Figure 5). Values are mean ± SD (n = 3 independent biological replicates). Different letters indicate statistically significant differences ($P < 0.05$, one-way ANOVA with Tukey's test).

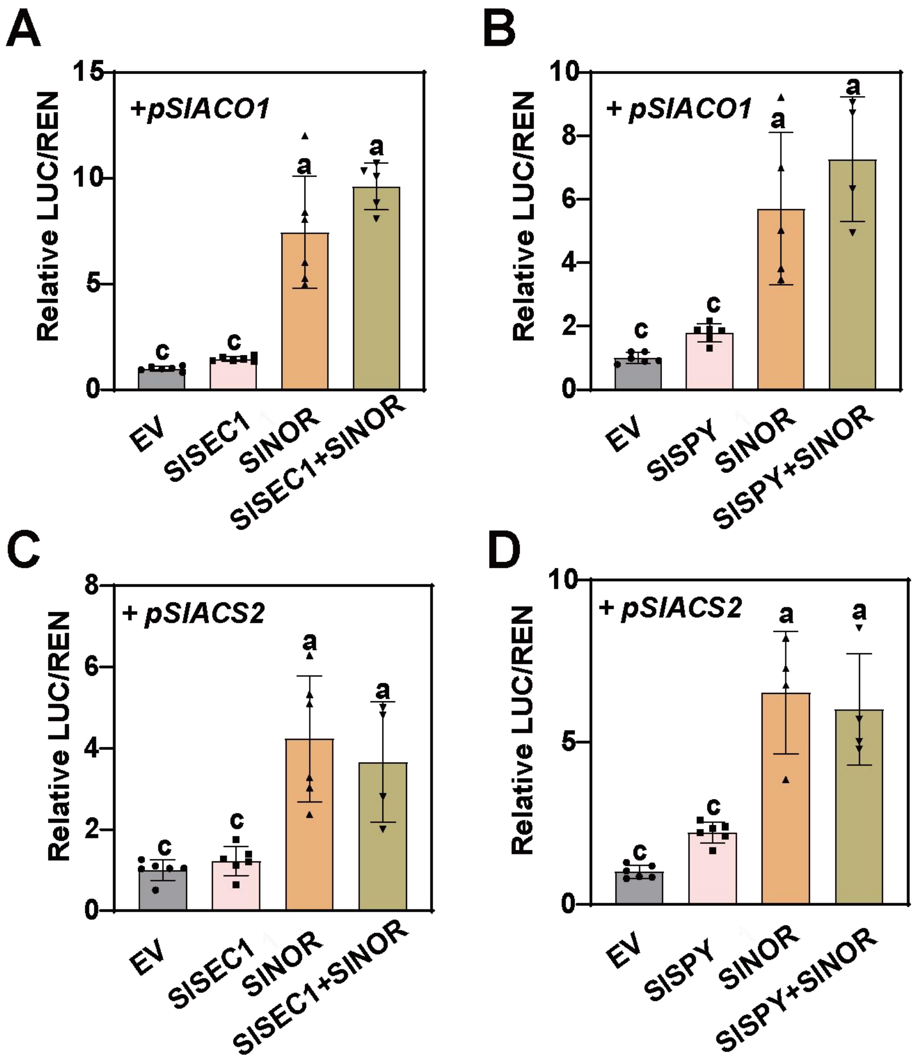

Supplementary Figure S11. Co-expression of either SlSEC1 or SlSPY with SlNOR does not enhance SlNOR-mediated transcriptional activation (Supports Figure 6). A–D) Dual-luciferase reporter assays showing the effect of co-expressing SlSEC1 or SlSPY with SlNOR on the *SlACO1* **(A, B)** and *SlACS2* **(C, D)** promoters in *N. benthamiana*. Empty vector (EV) is a control. Values are mean ± SD (n = 4 independent biological replicates). Different letters indicate statistically significant differences ($P < 0.05$, one-way ANOVA with Tukey's test).

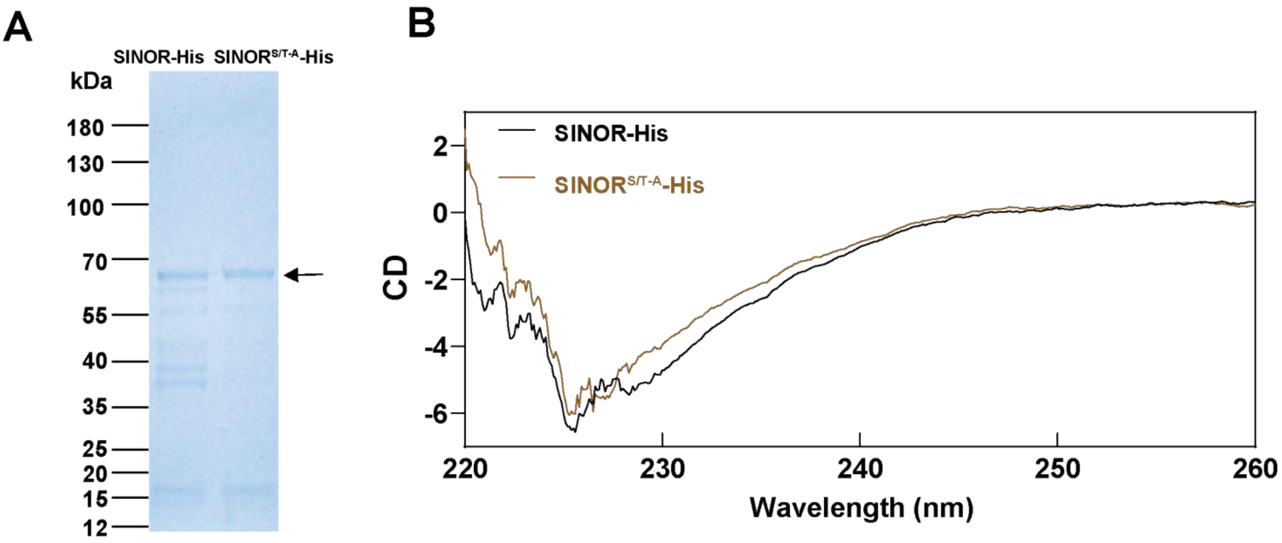

Supplementary Figure S12. The glycosylation-site mutation does not alter the secondary structure of SlNOR in circular dichroism (CD) assay (Supports Figure 6). A) Coomassie Brilliant Blue-stained SDS-PAGE gel showing comparable levels of purified SlNOR-His and the glycosylation-site mutant SlNOR$^{S/T-A}$-His. **B)** Far-UV CD spectra (220-260 nm) of purified SlNOR-His (black) and the glycosylation-site mutant SlNOR$^{S/T-A}$-His (brown).

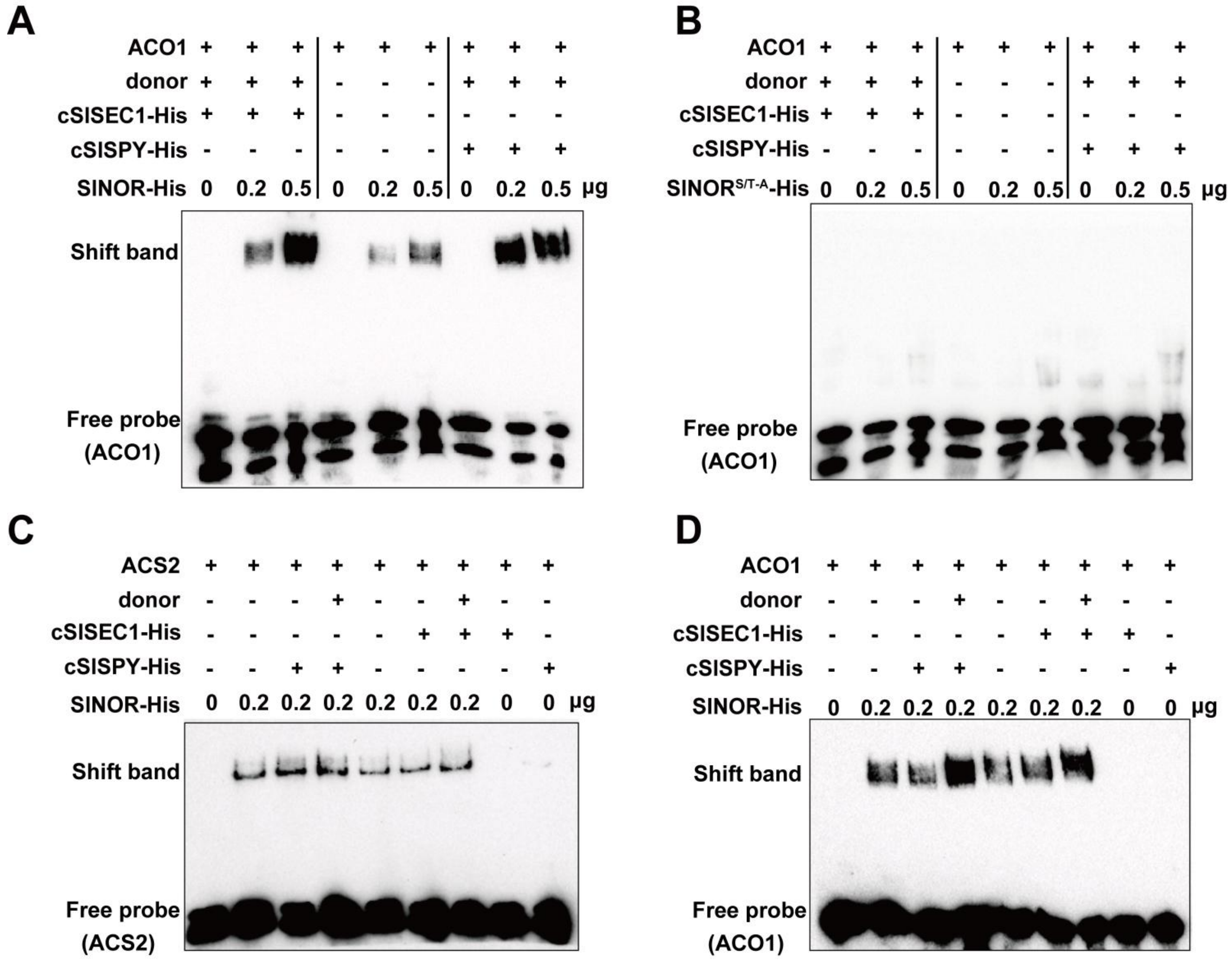

Supplementary Figure S13. *O*-Glycosylation mediated by SlSEC1 or SlSPY enhances the DNA-binding activity of SlNOR (Supports Figure 7). A, B) EMSAs showing DNA binding of *in vitro* glycosylated SlNOR-His **(A)** or SlNOR$^{S/T-A}$-His **(B)** to an *SlACO1* promoter probe. **C, D)** EMSAs showing DNA binding of SlNOR-His to *SlACS2* **(C)** and *SlACO1* **(D)** promoter probes upon *in vitro* glycosylation with cSlSEC1 or cSlSPY. Shifted complexes and free probes are indicated.
