# Supplementary material for: SlSEC1- and SlSPY-mediated O-glycosylation stabilizes the transcription factor SlNOR to promote tomato fruit ripening
Source: Plant Cell. 2026 May 14;38(6):koag144. doi: 10.1093/plcell/koag144 (PMC13274654; doi:10.1093/plcell/koag144)
Supplement: koag144_Supplementary_Data [file koag144_supplementary_data.zip › Supplementary Table.pdf]

**Supplementary Table S1. Gene model numbers of each species used in the phylogenetic tree.** All gene models and protein sequences were retrieved from Phytozome 14 (https://phytozome-next.jgi.doe.gov/) (Supports Supplementary Figure S2A).

| | **Gene model number** |
|---|---|
| *OsSPY* | LOC_Os08g44510 |
| *SlSPY* | Solyc09g010180 |
| *PtSPY* | Potri.006G208900 |
| *RcSPY* | 30032.t000025 |
| *VvSPY* | VIT_208s0007g02910 |
| *AtSPY* | AT3G11540 |
| *ZmSPY* | Zm00001d031938 |
| *SlSEC1* | Solyc09g097830 |
| *SlSEC2* | Solyc05g053835 |
| *OsSEC1* | LOC_Os02g28830 |
| *ZmSEC* | Zm00001d016506 |
| *VvSEC* | VIT_204s0043g00760 |
| *RcSEC* | 29680.t000092 |
| *PtSEC* | Potri.013G051400 |
| *AtSEC* | AT3G04240 |
| *OsSEC2* | LOC_Os01g68680 |

**Supplementary Table S2. Subcellular localization prediction of SlSEC1, SlSEC2 and SlSPY using DeepLoc2.1 (https://services.healthtech.dtu.dk/services/DeepLoc-2.1/) (Supports Supplementary Figure S7).**

| **Localization Probability** | **SlSEC1** | **SlSEC2** | **SlSPY** |
|---|---|---|---|
| Cytoplasm | 0.7298 | 0.7354 | 0.7117 |
| Nucleus | 0.4815 | 0.4646 | 0.4598 |
| Extracellular | 0.0417 | 0.0607 | 0.0461 |
| Cell membrane | 0.2991 | 0.3362 | 0.2555 |
| Mitochondrion | 0.1833 | 0.227 | 0.2341 |
| Plastid | 0.0283 | 0.0275 | 0.0206 |
| Endoplasmic reticulum | 0.2476 | 0.282 | 0.2085 |
| Lysosome/Vacuole | 0.2283 | 0.3168 | 0.1692 |
| Golgi apparatus | 0.3323 | 0.3249 | 0.2084 |
| Peroxisome | 0.2229 | 0.2142 | 0.2283 |
